# Evaluating the benefits of urban green infrastructure: Methods, indicators, and gaps

**DOI:** 10.1016/j.heliyon.2024.e38446

**Published:** 2024-09-25

**Authors:** Soheila Khalili, Prashant Kumar, Laurence Jones

**Affiliations:** a Global Centre for Clean Air Research (GCARE), School of Sustainability, Civil and Environmental Engineering, Faculty of Engineering and Physical Sciences, University of Surrey, Guildford, GU2 7XH, United Kingdom; b Institute for Sustainability, University of Surrey, Guildford, GU2 7XH, Surrey, United Kingdom; c UK Centre for Ecology & Hydrology, Environment Centre Wales, Deiniol Road, Bangor, LL57 2UW, United Kingdom; d Liverpool Hope University, Department of Geography and Environmental Science, Hope Park, Liverpool, L16 9JD, United Kingdom

**Keywords:** Urban green space, Co-benefits, Heat mitigation, Thermal comfort, Air quality, Sustainable development goals

## Abstract

Green infrastructure (GI) offers a promising solution for mitigating the adverse effects of climate change, but evaluating its effectiveness necessitates a comprehensive understanding of how that has been quantified in the literature. This study aims to review the methods (monitoring, remote sensing, and modelling) employed to assess the effectiveness of GI in urban areas for three ecosystem services: heat mitigation (cooling of air temperature), thermal comfort control, and air quality mitigation. The objectives include evaluating the suitability of these approaches across diverse scales, categorising the essential parameters, and identifying the strengths and limitations inherent in each method. Through a literature review, 126 research papers were selected for detailed analysis. Modelling was the dominant method for heat mitigation (45.6 %), thermal comfort (70 %), and air pollution (51.9 %). The main inputs for assessing these three ecosystem services by GI were: meteorological parameters used in monitoring or modelling, morphological parameters (describing vegetation, surface, and built-up area conditions), specified parameters depending on the evaluated benefit such as landscape metrics (for heat mitigation), personal factors (for thermal comfort), pollutant measures (for air pollution), and other parameters (e.g. building and traffic heat emissions). The application scale of each method was dependent on the instruments, satellite data, and simulation tools utilised. Monitoring methods were employed in studies ranging from street-scale to neighbourhood-scale, remote sensing methods covered city-scale to regional-scale assessments, and modelling studies spanned from street-scale to regional-scale analyses. These diverse methods used to assess the GI benefits each have individual strengths and limitations which need to match the context and objectives of the study.

## Highlights

• Reviewed scales, parameters, strengths, and limitations of GI assessment methods.
• More than half of the studies employed modelling approaches.
• Measurement duration in monitoring studies varied from one day to several months.
• Resolution of air pollution modelling studies varied from 0.4 m to 20 km.
• UTFVI, DI and UTCI used as thermal comfort indicators in remote sensing methods.

## Graphical abstract

**Figure:**
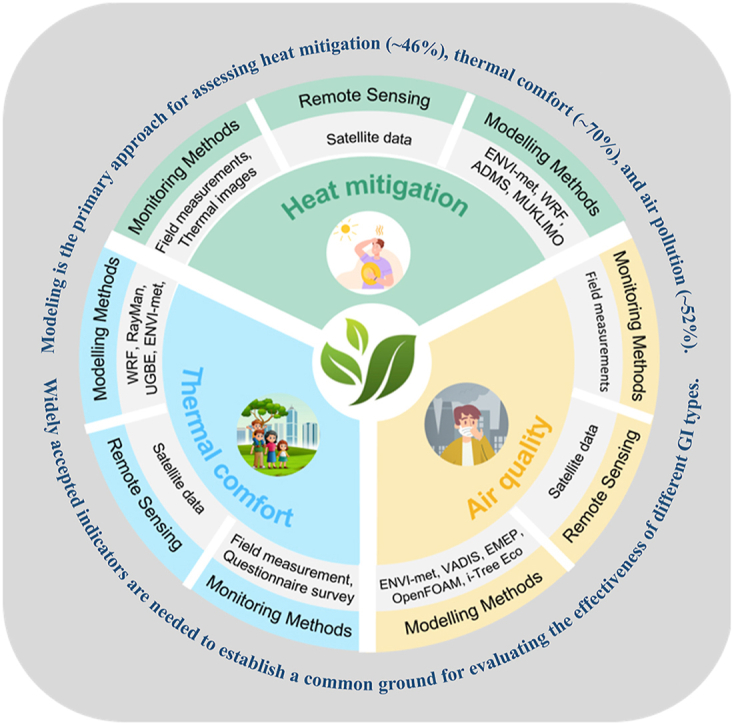

## 1 Introduction

The globally accelerated pace of urbanisation manifests in rising air pollution levels, increased temperatures, and compromised thermal comfort conditions in urban areas. These interrelated challenges pose severe threats to human health, ecosystem integrity, and urban sustainability [1,2]. Urban air temperature is strongly influenced by the built environment and is significantly warmer than its surrounding rural or peri-urban areas. Urban structures absorb solar heat (radiation) during the day and release it back into the environment at night. The overall health impacts of increasing temperature are negative but rarely receive adequate attention because the associated death tolls are not always immediately obvious [3,4]. The increasing temperature also has an adverse impact on thermal comfort conditions affecting public outdoor activities, tourism, health and well-being [5]. Moreover, air quality in the built environment continues to be a primary environmental issue as over half of the world's population currently lives in urban areas [6,7]. Exposure to air pollutants can prompt serious respiratory and cardiovascular health problems and increased mortality in the long term [8].

Green Infrastructure (GI) mediates a number of environmental functions which can reduce the harmful effects of climate change and to deliver a wide range of ecosystem services [9]. GI can significantly improve urban liveability and sustainability by reducing air pollution, mitigating the urban heat island (UHI) effect, and enhancing human well-being and biodiversity [7,10]. Evaluating GI performance is crucial for measuring its potential in delivering ecosystem services. Improving this knowledge base can help facilitate decision-making, and ensure the resilience and success of urban greening strategies. Various assessment methods can be applied to evaluate GI performance and the selection of the assessment methods directly influences the accuracy and reliability of the evaluation. These require alignment with the specific goals of the assessment process in both pre- and post-implementation phases of new GI [11]. Challenges include ensuring data accuracy, managing resource intensity, and defining the appropriate scale, parameters, and the required time for effective implementation and interpretation of results.

Previous review papers have extensively examined various dimensions of GI from different perspectives. For instance, Wang and Banzhaf (2018) focused on current GI mapping approaches at various scales and their associated functional analyses [12]. Zhang and Chui (2019) explored the benefits of GI for hydrological and biological systems [13]. The interplay between nature and human activity (Hansen & Pauleit, 2014) [14], the performance of informal GI [15], the GI effectiveness in indoor settings [16], and the effectiveness of different green-blue-grey infrastructure (GBGI) on mitigating urban overheating [7] were also discussed in past reviews. Hansen and Pauleit (2014) and Jones et al. (2022) evaluated the multi-functionality of GI [10,14], while Sharifi (2021) examined the synergies of urban climate change adaptation and mitigation [17]. Furthermore, Raymond et al. (2017) focused on policy and project implementation of GI interventions [18].

Other review papers have concentrated on specific benefits of GI, including air quality [[17], [18], [19], [20], [21], [22]], flood/stormwater management [[23], [24], [25]], heat mitigation [[26], [27], [28]], biodiversity [29,30], building energy use [16,31], cultural ecosystem services [32,33], human health and well-being [[34], [35], [36], [37]]. Reviews have also addressed planning and implementation of GI in urban areas [38,39] and in urban constructed heritage [40]. These studies employed various methods to estimate the effectiveness of GIs in different regions (Table 1).

**Table 1:** Summary of the most recent past review papers discussing multi-benefits, assessment methods, integration and implementation on GIs across hydro-meteorological hazards, air pollution, bio-ecological, health and well-being domains.

Article focus and key findings	Reference
● Carried out a systematic literature review on 202 evidence-based studies to analyse the effectiveness of different GBGIs covering 51 types across 10 categories. ● Efficient air cooling is found in botanical gardens, wetlands, green walls, street trees, and vegetated balconies. However, climate shifts may reduce their effectiveness due to changes in climate subtypes and zones in future.	[7]
● Conducted a systematic literature review based on 75 relevant articles on the quantifiable environmental, social, and economic benefits of both natural and engineered GI types. ● Investigated the existing knowledge trends and patterns, knowledge gaps, and served as a guide for estimating the benefits and challenges of GIs.	[41]
● Provided a synthesis of literature considering land cover, land use, and social and ecological functions for GI performance against a suite of urban issues. ● Offered an expert-led assessment tool for less-explored GI types enabling a-priori assessment of their potential contributions.	[10]
● Developed a guidance on quantitative pre-assessment of the potential co-benefits and disadvantages of Nature-based Solutions (NBS) addressing Disaster Risk Reduction. ● Discussed the evaluation of the quantified results on the pre-assessment, with a particular emphasis on assessing the significance of changes in estimated co-benefits and disbenefits.	[42]
● Examined scientific literature on monitoring methods for NBS performance against floods, droughts, heatwaves, landslides, and coastal hazards. ● Reviewed remote sensing techniques and explored challenges and prospects in monitoring NBS performance.	[43]
● Explored integration of NBS and preservation of urban heritage, addressing potential challenges. ● Highlighted NBS benefits for urban heritage like improved resilience, air quality, and heat reduction, noting obstacles such as conflicting priorities, limited funds, and public awareness.	[40]
● Established link between public urban green spaces and human well-being, showing consistent positive correlation with physical, mental, and social health. ● Identified factors influencing well-being and green space connection, encompassing space type, quality, local context, and individual traits.	[36]
● Addressed the co-benefits and synergies of urban climate change adaptation and mitigation measures, such as green spaces, sustainable transportation, and renewable energy technology.	[17]
● Emphasised the value of green spaces for enhancing both physical and mental health as well as for reducing the effects of climate change in urban settings. ● Focused on providing a ‘continuous’ connection with GI by providing thermally comfortable areas in order to get health benefits.	[35]
● Reviewed European research on GI and identifies its opportunities and challenges. ● Discussed the various ways that GI is defined and approached, the necessity of interdisciplinary research, and the significance of stakeholder involvement.	[44]
● Provided an update on nature-based solutions for hydro-meteorological hazards, including revised concepts, classification schemes, and databases. ● Aimed to improve understanding and facilitate the implementation of long-term strategies for mitigating the effects and risks associated with these hazards.	[45]
● Examined hydro-meteorological risk assessment methods and the role of nature-based solutions in managing flood, drought, and heatwave risks. ● Highlighted the importance of integrating these solutions into decision-making for effective mitigation of hydro-meteorological hazards.	[46]
● Emphasised research areas and bioecological benefits of GI practices across spatial scales. ● Established conceptual links between GI advantages for hydrological and bioecological systems at different scales.	[13]
● Conducted a review of 44 studies and showed that GI can bring benefits such as temperature regulation, improved air quality, and noise reduction in indoor settings.	[16]
● Proposed a conceptual framework for urban green infrastructure development focused on ecosystem services over multiple functions. ● Addressed the complex relationship between nature and human activities in urban environments.	[14]
● Introduced an ecosystem services framework to organise evidence on biophysical (e.g., CO_2_ sequestration) and socio-psychological (e.g., improved health) benefits. ● Provided framework aids in addressing climate change effects through coping (adaptation) or mitigation strategies.	[47]
● Analysed 200 studies on cooling effects of GIs, finding urban greening can reduce temperatures via shading and evaporative cooling. ● Examined temperature reduction from specific greenery types like parks, trees, green roofs, and ground vegetation.	[27]

Despite these extensive reviews, there is a lack of literature synthesising the methodologies used to assess GI benefits. There is a need for a comprehensive critique of existing methods and indicators, as well as a comparison of their applicability across different scales, the required parameters, and their strengths and limitations. Reviewing methodologies for GI evaluation provides an opportunity to understand the range of available tools and techniques, ensuring that the chosen method aligns with the specific goals of a given greening initiative. Given the diversity within studies in terms of scales, required parameters, and resources, this contributes to an improved understanding of the potential outcomes.

Therefore, the overall goal of this review paper is to assess the methods used to evaluate the benefits of GI in terms of heat mitigation (cooling of air or surface temperature), thermal comfort control, and improvement of air quality along with the associated indicators for evaluating the performance of GI, considering their strengths and limitations. The specific objectives of this review are to: (1) categorise the parameters used to assess GI performance for each benefit, (2) examine the duration and the scale of assessment methods, (3) evaluate the strengths and limitations of monitoring and modelling methods, (4) identify and highlight the research gaps in the assessment methods of GI benefits, (5) provide insights and recommendations regarding the application of different methods. Given the complex interplay between physical and physiological parameters of thermal comfort evaluation [48], thermal comfort and heat mitigation benefits are considered distinct benefits.

## 2 Scope, outline and methods

This study covers the monitoring, remote sensing, and modelling methods and the parameters employed in these methods for evaluating GI performance, as well as examining the scale and duration of these methodologies and identifying the specific GI types to which these methods have been applied. Given the focus on heat mitigation, thermal comfort, and air quality benefits, this review does not encompass other GI benefits related to social, health, economic aspects, and other environmental benefits. Moreover, the efficacy and performance of GI types are not within the scope of this paper. The reviewed methodologies are derived from papers that meet the search criteria.

The review focuses on a wide range of different GI types including green roof, green wall, trees, hedges, shrubs, forest, park, pocket park, public squares, grassland, and combinations of these GI types, based on the typologies of Jones et al. (2022) and Kumar et al. (2023) [7,10]. The final selection of specific GI types was based on their prevalence in the reviewed studies, following the method described below.

We carried out a literature review to identify, screen and refine peer-reviewed journals from Google Scholar, Web of Science, Scopus and ScienceDirect databases. The following keywords were used for the search: “green infrastructure” AND “heat mitigation”, “green infrastructure” AND “urban heat island” for heat mitigation benefit, “green infrastructure” AND “thermal comfort” for thermal comfort benefit, and “green infrastructure” AND “air quality”, and “green infrastructure” AND “air pollution” for air quality benefit to identify the relevant papers. Although numerous articles related to this research area have been published prior to 2010 the search was limited to peer-reviewed publications written in English from 2010 onwards, to only include the more up to date literature. Publications that discussed simulations that involved synthetic data or lacked information about specified locations or parameters were excluded.

**Fig. 1:**
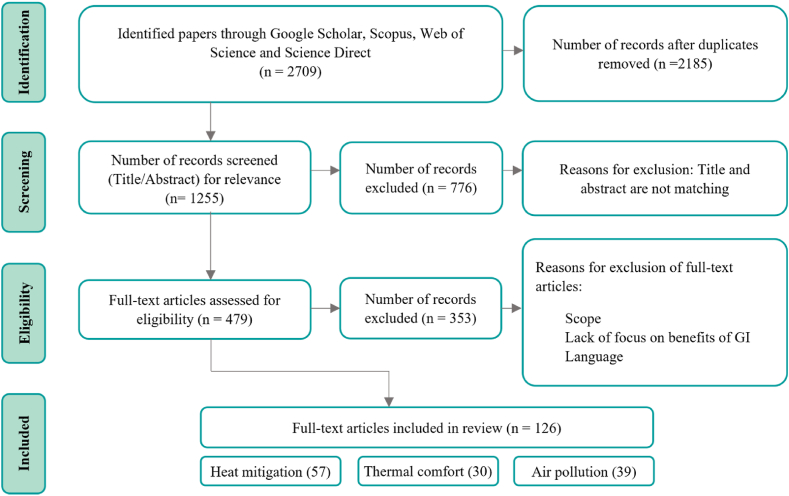
Schematic representation of papers identified in the steps of literature review.

Fig. 1 presents the steps used in identifying the relevant literature. After the identification and removal of 1255 duplicate papers, the dataset was refined to a total of 2185 studies for screening. Following the removal of 776 papers based on title and abstract, 479 papers were left for full-text screening. Finally, this process identified and included 126 relevant papers for inclusion. Most of the research papers were conducted in Asia (39.1 %), followed by Europe (33.3 %), North America (14.5 %), Australia (8.7 %), South America (2.9 %), and Africa (1.4 %). Among reviewed studies analysing GI heat mitigation benefit, green roofs were the focus in 25 % of the cases, followed by street trees (20.5 %). For studies on thermal comfort benefits, trees took precedence (30 %), followed by green walls and green roofs. Air quality benefits were predominantly studied in relation to hedges (25.9 %), green walls (22.2 %), and street trees (18.5 %). This review is comprised of seven sections. The structure of the paper is as follows. Section 1 introduces the background context and the need for this review, Section 2 describes the outline and scope of the paper. Section 3 describes the mechanisms by which GI benefits heat mitigation, thermal comfort, and air quality to build the background context relevant to monitoring and modelling methods. Section 4 focuses on the geographical distribution and the distribution of different methods and GI types. Then, the monitoring and modelling methods are discussed to provide an understanding of the scale and duration of their application, along with categorising parameters (Section 5), followed by conclusions and recommendations for employing GI evaluation methods (Section 6).

## 3 Mechanisms of heat mitigation, thermal comfort benefits, and air pollution mitigation by GI

### 3.1 Heat mitigation

GI reduces air and surface temperatures by evapotranspiration, shading, and increased albedo [49,50], thus resulting in decreased urban heating through a combination of these mechanisms. The evapotranspiration process uses solar energy to convert liquid water into vapour, replacing sensible heat with latent heat. As plants release water vapour and the soil moisture evaporates, they absorb and dissipate heat, acting as a natural cooling system that significantly reduces urban temperatures [7,51,52]. This reduction in sensible heat gain also helps lower the temperature of the plant canopy surface and decreases longwave radiation emitted into the surroundings. Qiu et al. (2017) investigated the relationship between evapotranspiration and UHI based on field measurement data to understand the mechanism of the cooling effect of GI. They employed the Bowen Ratio Energy Balance System to monitor the impacts of evapotranspiration on UHI. In this method, the Bowen ratio measuring system recorded meteorological data including T_a_, relative humidity (RH), precipitation, wind speed (WS), wind direction, soil heat flux, net radiation, solar radiation, and photosynthetically active radiation. They concluded that the evapotranspiration rate of 6.12 mm d^−1^ contributes to a 0.12 °C per hour decrease in UHI during the experimental condition [53]. Previous studies showed that for assessing green roof performance, soil moisture is a crucial factor as it determines evapotranspiration and it can be adjusted by an irrigation scheme in coupled Weather Research and Forecasting (WRF) and Urban Canopy Model (UCM) tools [54,55]. It is crucial to consider the specific characteristics of the GIs, such as vegetation type, density, and spatial distribution to accurately simulate their evapotranspiration effects in the context of heat mitigation.

Shading provided by the plant canopy is a highly effective way to cool urban microclimates. Depending on the density of their canopies, plants can intercept 70–90 % of incoming solar radiation, even reaching 50 % for deciduous trees in winter when they have fewer leaves [56,57]. This interception of both shortwave and longwave radiation significantly reduces the temperature of urban surfaces like buildings, roads, and pavements, consequently lowering the overall air and surface temperature. The effect of GI shading on heat mitigation can be evaluated through the canopy cover parameters such as tree crown characteristics, tree species, Leaf Area Index (LAI), and other factors which provide insights into the density and coverage of vegetation and comparison of the solar radiation or surface temperature in shaded and unshaded areas. Additionally, the GI can enhance the albedo of urbanised areas. For instance, built-up areas have albedo values ranging from around 0.1 to 0.2, while plants can have albedo values close to 0.3 [58]. Increasing albedo means more reflected incoming radiation, reducing the portion that gets absorbed and, consequently, lowering surface temperatures. The heat mitigation benefit measures provided by these mechanisms are discussed in Section S.1.

To effectively evaluate the performance of GI in reducing heat and inform future planning and design decisions, monitoring and modelling methods are employed. Monitoring involves the collection of real-time data on various environmental parameters (e.g., temperature, humidity, solar radiation, and wind patterns) to quantify the effectiveness of GI interventions. Concurrently, modelling techniques allow for the simulation of different scenarios, enabling researchers to predict the impact of GI on heat mitigation. Previous studies aiming to evaluate the cooling benefit of GI have employed monitoring, remote sensing, modelling or combined methods to measure the contribution of GI in reducing the harmful effect of UHI through different heat mitigation indicators. Section 5.1 details these different methods as well as the required input parameters.

### 3.2 Thermal comfort

Thermal comfort is defined as a subjective feeling of human satisfaction with the thermal environment [59]. Human psychological and physiological factors influence subjective perceptions. The main parameters to evaluate thermal comfort conditions are categorised into: (i) meteorological parameters including air temperature, relative humidity, wind speed, and mean radiant temperature, (ii) personal factors including clothing insulation, metabolic rate, age, health status, gender, body mass index, and adaptability [60].

The evaluation of outdoor thermal comfort conditions involves a combination of meteorological measurements, physiological models to model human responses to environmental conditions, and computational simulation tools to analyse complex thermal interactions. Additionally, human perception surveys and subjective assessments contribute to understanding how subjects perceive thermal conditions, all aiming to provide a comprehensive understanding of the interplay between environmental and human thermal comfort conditions in outdoor environments.

The meteorological parameters directly influence the thermal comfort conditions contributing to the convective, radiative, evaporative, and respiratory heat exchange between the human body and the surrounding area [61]. Previous research endeavoured to find the relationship between these parameters and thermal comfort conditions according to a 5-point scale varying from ‘very cold’ to ‘very hot’ that is defined as Actual Sensation Vote (ASV) or Thermal Sensation Vote (TSV) (Table S1).

A wide range of thermal comfort indices have been developed to assess human thermal comfort conditions. These models are categorised into mechanistic and empirical models based on the way in which they have been developed; the mechanistic models which are based on human thermal balance can also be divided into equivalent temperature and thermal load models [61]. In this review, we focus on the equivalent temperature models, which are the most widely employed method in outdoor thermal comfort studies. The equivalent temperature refers to the air temperature of an indoor room setting that provides the same physiological responses, such as skin and core temperatures, skin wetness, and other reactions as the actual complex conditions [61]. The common outdoor thermal comfort models including PET, UTCI, and outdoor Standard Effective Temperature are based on equivalent temperatures [62] which are discussed in Section 5.2.

Thermal comfort conditions in the urban environment are complex and cannot be explained merely by air temperature [63]. Previous studies indicated that Mean Radiant Temperature (MRT) and wind speed are significantly effective factors influencing outdoor thermal comfort [64], as well as air temperature, which has the highest impact on outdoor thermal comfort conditions [[65], [66], [67]].

GI plays a vital role in enhancing thermal comfort by mitigating heat, changing wind speed, and reducing direct sunlight exposure resulting in lowering the MRT. Some studies focused on the effect of GI types on the magnitude of solar radiation and MRT levels, reporting the effect of GI as shading structures resulting in the improvement of thermal comfort conditions [68]. Moreover, other studies have reported the effect of GI on wind speed and ventilation affecting thermal comfort conditions [69]. GI has also an effective influence on thermal comfort by reducing the frequency of severe thermal sensation conditions and increasing the thermal ‘neutral’ condition [58]. The thermal comfort benefit measures provided by GI are discussed in Section S2.

### 3.3 Air quality

Urban air pollution in many cities around the world is reduced by GI. Previous studies have developed dynamic modelling approaches to assess the role of GI in air pollution by accounting for chemical interactions between pollutants, meteorological conditions, and pollutant dispersion. Microscopic particles, such as PM_2.5_ and PM_10_ can deeply penetrate the respiratory system and affect human health, with the reduction of PM_2.5_ concentrations offering the most significant health benefits [[70], [71], [72]]. Other pollutants include NO_2_, O_3_, and CO, each contributing to adverse health effects, such as smog formation and respiratory system irritation. Additionally, the organic compounds called bVOCs can combine with other pollutants to create particulate matter and ground-level ozone [73].

Urban vegetation reduces pollutants through enhanced dispersion and deposition [[74], [75], [76], [77]]. The quantities of pollution removal are influenced by the planting design, plant characteristics [73] and other meteorological factors that influence tree transpiration and deposition velocities [74]. Air pollution is a complicated mixture of nano- and micro-sized particles as well as gaseous pollutants. The air quality indicators are categorised into particulate matter of various size ranges (PM_10_, PM_2.5_), ultrafine particles (UFPs), and gaseous pollutants (O_3_, NO_2_, SO_2_, and CO) in which exposure to these pollutants has been linked with respiratory and cardiovascular diseases [78]. The measures provided by GI for enhancing air quality are explored in Section S3.

To assess the air quality improvement provided by GI, different methods include deploying sensors for real-time pollutant measurements and modelling approaches which employ computational simulation and GIS analyses to evaluate the impact of GI interventions. The methods and parameters required for assessment are discussed in Section 5.3.

The net benefits for some pollutants are small and can even increase pollutant concentrations locally through chemical interactions depending on the tree species selection [79]. The interaction between air pollutants and the design of green infrastructure (such as species selection and spatial placement) can either improve or deteriorate individual exposure and consequently human health [19,20].

Furthermore, GI provides interconnected benefits through common mechanisms. GI mitigates heat through evapotranspiration and shading, reducing ambient and surface temperatures, and thereby improving thermal comfort conditions. Additionally, wind modulation by GI mitigates heat, enhances thermal comfort, and helps disperse pollutants.

## 4 Types of GI benefit assessment methods

**Fig. 2:**
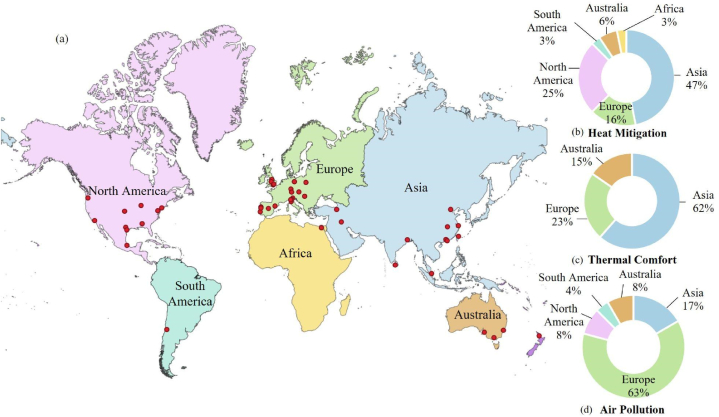
(a) Geographical distribution of reviewed papers based on their location (latitude and longitude). The pie chart shows the distribution of GI benefits studies in terms of (b) heat mitigation, (c) thermal comfort, and (d) air pollution by continent.

The geographical distribution of studies on GI benefits in terms of heat mitigation, thermal comfort, and air quality is shown in Fig. 2. The results show that most of the studies focusing on heat mitigation (47 %) and thermal comfort (62 %) were carried out in Asia as opposed to most of the reviewed studies on the air quality benefit of GI in Europe (63 %).

Modelling methods emerged as the most prevalent approach for analysing heat mitigation (45.6 %), thermal comfort (70 %), and air pollution (51.9 %). Subsequently, mixed methods which encompass a combination of modelling, monitoring, and remote sensing approaches emerged as the second most widely used method for both heat mitigation (22.7 %) and thermal comfort (20 %) studies. Remote sensing for heat mitigation was employed by 22.7 % of the total studies; the search outcomes showed that this method was not widely used for the assessment of thermal comfort benefit which can be attributed to the limitations of low temporal resolution for high spatial resolution and vice versa in remote sensing methods (Wu et al., 2014) that are vital factors for assessment of thermal comfort conditions at the human scale. Monitoring methods are mostly adopted for air pollution studies (33.3 %), followed by heat mitigation (18.2 %), and thermal comfort (10 %) studies.

The scale of the assessment domains (different from the resolution of the data) via monitoring, remote sensing, and modelling methods exhibited considerable variability, ranging from 1.2 × 10^−4^ ha (street-scale) [80] to 8.8 × 10^6^ ha (regional-scale) [70]. The scale varied from street to neighbourhood for in-site measurements and from city to regional in most of the remote sensing studies. Modelling studies employed assessment scales spanning from 0.07 ha [81] to 8.8 × 10^6^ ha [70] covering all street, neighbourhood, city, and regional scales. The assessment scale is an influential factor in defining the resolution of the modelling studies. Among the reviewed studies, the resolution of modelling studies varied widely from 0.4 m [82] to 20 km [83], depending on the research objectives, model configuration, assessment scale, and grid size. Each of the monitoring, remote sensing, and modelling methods includes different categories with specific applicability scale and duration of measurement. Table 2 presents a categorisation of various methodologies, described by their characteristics, the scales at which these methods are applicable-ranging from street to regional scale and the duration of measurements which varies from short-term (days) to long-term (years).

**Table 2:** An overview of various methodologies for GI benefits assessment categorised by characteristics, applicable scale, and measurement duration.

Methodology	Category	Characteristics	Scale	Duration of measurement
Monitoring	Field measurement	Direct, on-site real-time data collection	Street and neighbourhood scale	Days to months
	Scaled model measurement	Physical models used to simulate real conditions	Street and neighbourhood scale	Days to months
Remote sensing	Thermal Infrared (TIR)	Measures land surface temperature	City and regional scale	Periodic measurement
	Multispectral and Hyperspectral	Captures data across multiple spectral bands; land cover and greenery analysis	City and regional scale	Periodic measurement
	Lidar (Light Detection and Ranging)	High resolution 3D data	City scale	Periodic measurement
	Optical and Near-Infrared (NIR)	High-resolution imagery for vegetation and surface characteristics	City and regional scale	Periodic measurement
	Low-Altitude thermal infrared	High-resolution imagery for vegetation and surface characteristics	Street and neighbourhood scale	Days to months
Modelling	Empirical models	Based on observed data and statistical analysis	Various scales	Periodic measurement
	Process-Based models	Simulate physical and chemical interactions	Various scales	Periodic measurement
	Geospatial models	Utilise GIS data; suitable for mapping and spatial analysis	Various scales	Continuous monitoring
	Simulation models	Algorithms and mathematical models	Various scales	Depends on simulation configuration
Hybrid		Combination of multiple methods	Various scales	From days to years

The duration of assessment in the reviewed studies was highly dependent on the objectives of the study. The duration of studies among the reviewed studies was up to 7 months for the in-site measurements [84], up to 20 years for remote sensing studies [85], and up to one year covering all seasons for modelling studies with from 4 to 24 h spin-up time [70,86]. The minimum duration of the assessment was one day across all methodologies [87].

## 5 Monitoring, remote sensing, and modelling methods for assessing the GI benefits

### 5.1 Heat mitigation

#### 5.1.1 Indicators

Urban heating is measured using indicators derived from (a) the stationary and mobile measurements measuring T_a_ or (b) the satellite-based measures of land surface temperature (LST) [88]. These two methods are used to analyse the intensity of UHI, which can be categorised into three main types: Surface Urban Heat Island (SUHI), Boundary Layer Heat Island (BLHI), and Canopy Layer Heat Island (CLHI). SUHI is measured using LST and offers spatial coverage and highlights variation in land surface cover (e.g. built land, trees, grass, waterbodies, etc.). BLHI and CLHI are measured using T_a_, reflecting the thermal properties of the boundary layer and the temperatures within the urban canopy layer, respectively. Studies comparing UHI intensity measured using T_a_ and LST found that the results depend on the chosen indicator and data used [89]. The surface energy balance during the daytime mainly controls the difference between the T_a_ and LST [90]. The ground-based T_a_ method provides precise real-time measurements, providing a more accurate reflection of the "perceived temperature" associated with human health [90]. In contrast, LST is a modelled product derived from satellite data that provides continuous spatial coverage, whereas point measurements of T_a_ lack spatial continuity and do not account for surface land cover variations [91].

A wide range of studies showed that GI effectively mitigates urban heating, as indicated by changes in ground-based and satellite-based measurements before and after GI implementation. They employed different monitoring, remote sensing, and modelling methods based on the objectives, resources, scale, strengths, and limitations of the assessment methods, and other factors. Consequently, it is important to assess different methods employed in previous studies and the required parameters used for analysing the heat mitigation benefit provided by GI.

#### 5.1.2 Monitoring methods

Monitoring procedures to measure the heat mitigation provided by GI directly utilise various sensors at different heights to measure meteorological parameters using stationary weather stations and mobile measurements (Table 3). The height of sensor installation varied between 0.15 m and 1.7 m (Table 3). While some studies set the sensors at 1.1 or 1.2 m representing the typical adult body's balance point, most of the studies consider a height of 1.5 m to represent the pedestrian level. The temporal period of the studies ranged from one day to three months. Most of the monitoring studies were conducted on street-scale GI interventions but some of them were carried out in urban parks representing neighbourhood-scale.

**Table 3:** Summary of previous studies and derived information of heat mitigation monitoring (the used instruments, temporal period, measurement height, and scale) and modelling studies (simulation tools, domain and grid size, resolution, and scale).

Monitoring^a^					
Instruments	GI type	Duration of measurement	Measurement height	Scale	Reference
Kestrel NK-5500	Vertical greening, traditional greening, quality improvement greening	One day (September) 10:00 to 14:00	–	Street-scale	[87]
HOBO U12, TESTO 480, CNR4 Net Radiometer (with LogBox SE), LI-COR	Green roof, green wall, ground tree	Four days from 9:30 to 17:25	1.5 m above GL^c^ and 1.5 m above the green roof	Street-scale	[92]
Kestrel 5400	Extensive/Intensive green roofs, green façade, street tree	Six days in October and April	1.5 m above GL	Street-scale	[93]
Fluke 971	10 large urban parks	Ten days in July and August at 13:00 and 20:30 (each run less than 80 min)	1.2 m above GL	Neighbourhood-scale	[94]
HOBO (S-THB, TMC-HD) Infrared radiometer (SI-111, Apogee, Logan, UT) Net radiometer (CNR4, Kipp & Zonen, Delft)	Green Roof	3 months	0.15, 0.5, and 1.5 m above the green roof	Street-scale	[95]
Onset HOBO (S-THB, S-WCA, S-LIB, S-RGB), LSI (EST)	Trees	Nine days in summer season (3 consecutive days in each month)	1.1 m above GL	Street-scale	[96]
HOBO (H21, U12)	Green wall	Two sets of measurement for 99 and 70 days considering 5 and 9 days out of them for analysis	1.7 m above GL	Street-scale	[97]
HOBO (S-THA, S-LIB, S-WSA, S-WCA)	Pocket parks	Three days in May and July from 1 to 10 p.m.	–	Street-scale	[98]
TESTO 545, TESTO 625, TESTO 845	Green wall	April to September, weekly at 14:00	–	Street-scale	[99])
WatchDog Model 2550	Trees	Nine days in July and August- four pair of measurements on each day from 10:00 to 14:00	1.5 m above GL	Neighbourhood-scale	[100]
Remote sensing					
Satellite	GI type	Duration	Description	Scale	Reference
Landsat 8 OLI-TIRS	Urban green landscapes	One day (September)	Spatial resolution of 130 m	City-scale	[101]
Landsat 8 OLI-TIRS	Parks	Summers of the years 2014–2018	Spatial resolution of 100 m	City-scale	[102]
Landsat 5 TM and Landsat 8 OLI-TIRS	Urban green space	3 years (February)	Spatial resolution of 100 and 120 m	Regional-scale	[103]
Landsat 5 TM, Landsat 7 ETM+, and Landsat 8 OLI-TIRS	Urban greenery	20 years	Spatial resolution of 60 m, 100 m, and 120 m	City- scale	[85]
Modelling^b^					
Simulation tool	GI type	Model description		Scale	Reference
WRF	Different GI fractions	- Over a parent domain and two nested domains with spatial resolutions of 18 km, 6 km, and 2 km - Grid distance (km): 30, 6, 2 - Grid number: 120 × 120, 206 × 206, 154 × 154 - Number of vertical layers: 33 layers		City-scale	[104]
	Different vegetation patches and fractions	- Over a parent domain and two nested domains with spatial resolutions of 18 km, 6 km, and 2 km - Grid distance (km): 18, 6, 2 - Grid number: 120 × 120, 206 × 206, 154 × 154 - Number of vertical layers: 33 layers		City-scale	[105]
	Increasing vegetation area: moderate and intensive scenarios	- Four two-way nested domains with 37 × 22, 43 × 34, 91 × 61, and 145 × 91 grid points in east-west and north-south directions. - Horizontal resolution of 9, 3, 1, and 0.333 km - Vertical resolution within 51 eta level^d^		City-scale	[106]
	Mixed forest, combined mixed forest and grasslands, Combined mixed shrublands and grasslands	- Three nested domains at 18, 6, and 3 km horizontal resolution. - Number of vertical layers: 38		City-scale	[107]
ENVI-met	Vegetated surfaces and trees	- Model domain: 960 × 960 × 40m - Grid size: 160 × 160 × 20 - Resolution: 6 × 6 × 2m		Neighbourhood-scale	[108]
	Vertical greening, traditional greening, quality improvement greening	- Model domain: 1000 × 910 × 45m - Grid size: 200 × 182 × 15 - Resolution: 5 × 5 × 3m		Neighbourhood-scale	[87]
	Extensive/Intensive green roofs, green façade, street tree	- Model domain: 250 × 250 × 200m - Grid size: 125 × 125 × 100 - Resolution: 2 × 2 × 2m		Street-scale	[93]
	Pocket Park, façade and roof greening	- Model domain: 420 × 330m - Grid size: 84 × 66 - Resolution: 5 × 5m		Neighbourhood-scale	[109]
	Street trees	- Model domain: 90 × 90 × 30m - Grid size: 90 × 90 × 30 - Resolution: 1 × 1 × 1m		Street-scale	[69]
	Different combinations of green roof, green wall, and plantation	- Model domain: 150 × 170m - Grid size: 30 × 34 - Resolution: 5 × 5m		Street-scale	[110]
	Greenway, green roof, grove	- Model domain: 300 × 160 × 60m - Grid size: 150 × 80 × 30 - Resolution: 2 × 2 × 2m		Street-scale	[111]
	Trees and grass, green roof	- Model domain: 180 × 180m - Grid size: 90 × 90 - Resolution: 2 × 2m		Street-scale	[112]
ADMS-TH	Green roof, grassland, trees	- Domain size: 9.5 × 8 km - Resolution: 10 × 10 m		City-scale	[113]
		- Domain size: 2 × 1.8 km		Neighbourhood-scale	[114]
MUKLIMO-3	Trees, grassland	- Model domain: 20 × 17 km - Horizontal resolution: 100m - Vertical resolution: from 10 to 100m		City-scale	[115]
Hybrid					
Method	GI type	Duration	Description	Scale	Reference
Field measurement/questionnaire survey/ENVI-met simulation	Vertical greening	One day (September)	- Grid size: 200 × 182 × 15 - Resolution: 5m horizontal and 3 m vertical	Neighbourhood-scale	[87]
Field measurement/ENVI-met simulation	Street trees	−Two days (January and August)- representing coldest and hottest days of year	- Grid size: 80 × 80 × 30 - Resolution: 1m	Street-scale	[69]
ENVI-met simulation/Satellite data	Green roof and green wall	- One day	- Satellite data resolution: 15–30 m - Model resolution: 5m - Grid size: 150 × 170	Street-scale	[110]

a Typical instruments used for heat monitoring include thermal cameras, Kestrel, Fluke, Testo, and HOBO weather stations and data loggers.

b Typical simulation tools used for heat mitigation modelling include ADMS, ENVI-met, WRF, MUKLIMO-3, and UCM models.

c GL: Ground Level.

d The eta level is calculated by (P-P_T_)/(P_S_-P_T_), where P is the dry hydrostatic pressure at each corresponding level, P_S_ is dry hydrostatic surface pressure, and P_T_ is a constant dry hydrostatic pressure at the top of the model.

#### 5.1.3 Remote sensing methods

Remote sensing plays a pivotal role in dissecting the intricate relationships between LST and vegetation coverage at city and regional scales. The diversity of satellite platforms employed in previous studies underscores the importance of selecting appropriate data sources, which influences the accuracy, scale, and applicability of the results.

Several studies have utilised a range of satellite data to retrieve LST and evaluate the effect of GI on its reduction. For instance, Landsat 8 OLI/TIRS was utilised for LST retrieval on cloud-free days [[116], [117], [118],]. Sentinel-2 multispectral instruments were used to analyse 2D land surface characteristics such as Normalised Difference Vegetation Index (NDVI), Normalised Difference Built-up Index (NDBI), Leaf Area Density, and LAI. Additionally, LiDAR point clouds provided data on 3D urban morphology parameters, including building roof index, building volume density, sky view factor, solar radiation, building height [[119], [120], [121]]. Considering the variations in radiative temperature between ground surfaces and the vegetation canopy, the majority of remote sensing studies have focused on investigating the thermal fluctuations of vegetation cover through linear correlations. To obtain surface reflectance values, Lemoine-Rodriguez et al. (2022) pre-processed the satellite images using a Dark Object Subtraction image-based atmospheric correction to the visible and infrared bands to calculate the surface reflectance values employing the R package [121]. Gao et al. (2022) ran the LST retrieval method in Python and parameterized their calculated park cooling effect using a Gaussian model [102].

The scale of reviewed remote sensing studies varied from 6.5 × 10^7^ m^2^ [121] to 8.9 × 10^8^ [116] representing city and regional-scales. Buffer zones are crucial for quantifying the extent and cooling impact of GI in the surrounding urban environment. Applying buffer zones and investigating the LST within them contributes to establishing the LST-distance relationship and quantifying the park cooling intensity using different methods including the fixed radius method, first turning point, equal radius method, and the equal area method [116,120]. The size and configuration of buffer zones are critical variables affecting the study outcomes. While many studies apply 10–20 buffer zones, each 30 m wide, Gao et al. (2022) used six 90-m wide buffers, highlighting the variability in methodological approaches and the potential impact on findings [102]. Additionally, other studies have used buffer widths based on the length of the park radius [122,123].

The temporal scale of studies varies, with some studies based on single image for a one-year study to 20 images for a five-year study [116,120,102]. The frequency and timing of image acquisition are critical factors that influence the temporal resolution and the ability to detect seasonal and interannual variations on LST.

#### 5.1.4 Modelling methods

The effectiveness of GI on heat mitigation through simulating different scenarios can be evaluated using several simulation tools such as the WRF model, ENVI-met, ADMS-TH, and MUKLIMO-3 which are discussed in the following paragraphs. The WRF model provides several physics options for each physical parameterization, including the Land Surface Model, Planetary Boundary Layer, cumulus parameterization, short and longwave radiation, and microphysics [[124], [125], [126]]. In order to consider detailed canopy processes, a single-layer Urban Canopy Model has been widely coupled with WRF to simulate the heat mitigation strategies for urban heating reduction [107,111]. UCM determines the fluxes for the urban surfaces within a grid cell and includes parameterization of physical processes involved in heat exchange, momentum, and water vapour by incorporating reflections, shadowing, radiation trap, surface energy budget of the built environment, and anthropogenic heat emissions [24,54]. Studies employed WRF to evaluate the effect of increased fractions of vegetated patches per grid cell from 20 % to 100 % at the city-level scale for assessing mitigation potential and urban climate impact. Based on the previous studies the first 24–72 h of the WRF simulation were taken as model spin-up time and were discarded from post-processing [107,105,104].

ENVI-met is a comprehensive, three-dimensional simulation tool for modelling urban microclimates. Developed by Michael Bruse in 1994, this Soil-Vegetation-Atmosphere Transfer (SVAT-) type model integrates soil, vegetation, and atmosphere, setting it apart from other climatic simulation programs. In reviewed studies, various scenarios including green walls, street trees, intensive and extensive green roofs, and different percentages of a combined green walls and green roofs scenario were simulated using ENVI-met modelling [[93], [109], [110], [127], [128]]. In order to ensure a stable numerical simulation and to minimise the edge effect in ENVI-met simulation, a typical nesting grid of 5–10 cells has been considered [127,109,129].

ADMS-TH is part of the Atmospheric Dispersion Modelling System by Cambridge Environmental Research Consultants for assessing GI's impact on heat mitigation. This simulation tool generates high-resolution heat maps using land cover data and urban fabric details. The model accurately estimates temperature changes from both surface types and anthropogenic heat sources in urban areas [130]. Tiwari et al. (2021) utilised ADMS-TH modelling to compare the existing scenario with five new scenarios including implementing GI, non-GI scenario, maximum green roofs, maximum grassland, and maximum trees to evaluate the suitable mitigation strategy [113].

Mikroskaliges Urbanes KLimaMOdell 3-dimensionale (MUKLIMO-3) is a mesoscale model. It was created as an urban climate model to replicate the near-surface meteorology of urban areas. The model can offer atmospheric data (such as temperature fields) at a high spatial resolution, which is essential for urban climate analysis, and it can also simulate airflow fields in the presence of buildings. Simulation times range from a few hours to several days, and typical spatial domains are 750 m high, 25 km wide, and 100 m long, with horizontal resolutions [131]. MUKLIMO-3 can be coupled with the microscale model ENVI-met, via Huttner's (2011) offline dynamic down-scaling technique [131]. Gal et al. (2021) carried out an urban scale simulation with MUKLIMO-3 microclimatic model using a Local Climate Zone (LCZ) map to identify the heat mitigation of dense trees, scattered trees, grassland, and emission scenarios for future climate [132].

Each of the simulation tools has some strengths and limitations, so it is important to select the appropriate simulation tool based on the evaluation objectives and context. The WRF model works with resolutions in kilometres, since the impacts of GI are often localised, the model may not capture small-scale variations in land use and surface characteristics. ENVI-met has limitations, such as constraints in modelling large areas due to computational demands and simplification of geometries to cuboid shapes. Large-area modelling necessitates larger grid cell sizes, which lowers input and output accuracy affecting the resolution of the results. Furthermore, ENVI-met is unable to simulate anthropogenic heat or the heat produced by human activity. Nonetheless, its capacity to simulate urban climate processes and estimate effects, like temperature variations in both horizontal and vertical domains, makes it a pivotal tool for assessing the impacts of urban green infrastructure on microclimates and energy conservation.

ADMS-TH boasts a shorter run time compared to peers, enhancing efficiency in scenario evaluations [133]. It calculates temperature and humidity perturbations based on local spatial variation and its methodology addresses surface moisture changes and shear stress perturbations, offering a comprehensive simulation of heat dispersion. This simulation tool can be broadly utilised at different scales varying from neighbourhood/street scale [134] to city-scale. The MUKLIMO-3 simulation tool spatial resolution can range widely, from several hundred metres [135] with parameterized building settings to a few metres with resolved buildings, but this tool does not consider some variables such as the cloud processes and precipitation [136] and the model output is limited to the absolute air temperature and relative humidity.

The reviewed studies on modelling the heat mitigation benefit of GI were conducted on scales ranging from city-scale, neighbourhood-scale, to street-scale depending on the employed simulation tool, providing insight to select the best simulation tool based on the scale of assessment and objectives. The resolution of the modelling is an important factor depending on the required details of the assessment with the resolution varying from 1 m to 18 km in the reviewed studies (Table 3). The selection of an appropriate resolution is critical to ensuring that the model accurately represents the complexities of the investigation site while also aligning with the specific objectives and scope of the assessment.

**Fig. 3:**
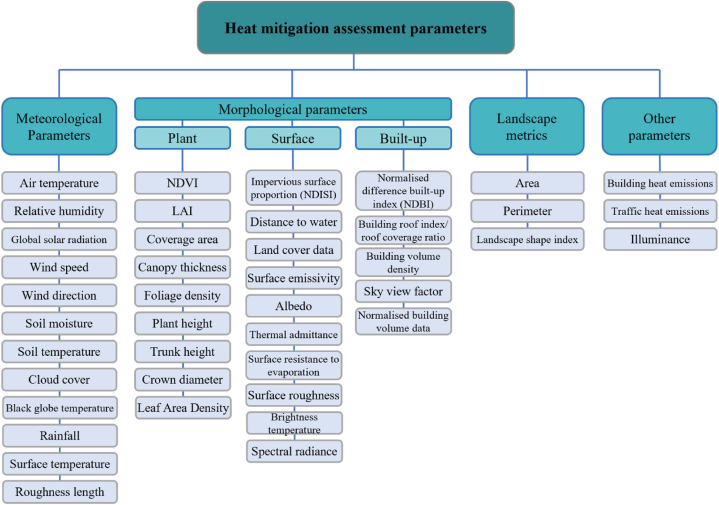
A set of parameters derived from previous papers for monitoring, remote sensing, and modelling the heat mitigation benefit provided by GIs at various scales.

The parameters used for assessing the heat mitigation benefit provided by GI in the reviewed studies through monitoring, remote sensing, and modelling methods are categorised into 1) meteorological parameters, 2) morphological parameters including plant, surface, and built-up area characteristics, 3) landscape metrics, and 4) other parameters (Fig. 3). These parameters can be measured using field measurements, stationary databases, field surveys, satellite data, and other sources. The parameters are variously used for assessing the GI benefits and as inputs for simulation tools to estimate the heat mitigation benefit provided by GIs.

### 5.2 Thermal comfort

#### 5.2.1 Indicators

To assess and quantify thermal comfort conditions, researchers have developed different thermal comfort indices. These indices aim to provide a standardised measure that incorporates multiple parameters and factors affecting human thermal perception. Among different thermal comfort indices UTCI and PET have been widely used in previous studies to assess thermal comfort conditions for outdoor environments. The PET is based on the Munich Energy-Balance model for individuals [137] and the UTCI is based on the multi-node dynamic thermophysiological UTCI-Fiala model [138]. The UTCI combines the thermophysiological impacts of T_a_, MRT, RH, and v. It includes an adaptive clothing algorithm, designed to respond to climate conditions [139]. The numerical model at the core of UTCI envisions an individual walking at 4 km/h on level ground, representing a metabolic rate of 135 W/m^2^ (2.3 MET).

#### 5.2.2 Monitoring methods

This section focuses on the different monitoring methods employed to assess the thermal comfort improvement by GI. It explores the use of field measurements and various sensors for measuring the factors which influence outdoor thermal comfort conditions. Table 4 provides a summary of some previous studies and measurement details in outdoor thermal comfort evaluation.

**Table 4:** Summary of previous studies and derived information of thermal comfort monitoring (instruments, temporal period, measurement height, and scale) and modelling studies (on simulation tools, domain size, grid size, resolution, and scale).

Monitoring^a^					
Instruments	GI type	Duration of measurement	Measurement height	Scale	Reference
AM-101 PMV + questionnaire survey	Trees, shrubs	Four days: 17, 18, 20, 2 July	1.1 m above ground GL∗	Street-scale	[140]
JT-IAQ-50	Green wall	One day (July) 9:00 to 17:00	1.5 m above GL	Street-scale	[141]
Hanwell ML4109	Green roof, green wall	Between June and September 2019	1.8 m above GL	Street-scale	[142]
Testo, JTR-04/05, TENMARS TM404	Park	Two days in summer from 9:00 to 18:00 and three days in winter from 7:00 to 20:30	1.5 m above GL	Neighbourhood-scale	[143]
Kestrel 4400	Trees	Two weeks (June and July) 12:00 to 14:00	1.1 m above GL	Neighbourhood-scale	[144]
HOBO MX2301A, Proster Digital Anemometer MS6252a + questionnaire survey	Park	Six days between June and September	1.5 m above GL	Neighbourhood-scale	[145]
Graphite-midi GL820+ questionnaire survey	Green wall	Eighteen days from September to November (48 h each site)	2.0 m above GL	Street-scale	[146]
Campbell CS215L, Gill windsonic	Street trees, front gardens	Five days between July and August- from 9:00 to 16:00	1.2 m above GL	Street-scale	[147]
Remote sensing					
Satellite	GI type	Duration	Description	Scale	Reference
Landsat 5 and Landsat 8 OLI/TIRS	Urban vegetation cover	1989, 1999, 2009, and 2019	Spatial resolution of 30 m	City-scale	[148]
Landsat 5 TM and Landsat 8 OLI/TIRStral	Urban greenery	2011 and 2019	Spatial resolution of 30 m	City-scale	[149]
Landsat 8 OLI/TIRS	Green roof	Two days (January and August 2016)	Spatial resolution of 30 m	City-scale	[150]
Modelling^b^					
Simulation tool	GI type	Model description		Scale	Reference
WRF + RayMan	Urban greening	- Three nested domains at 4.5, 1.5, and 0.5 km horizontal resolution.		Regional-scale	[151]
	Green roofs	- Two way nested domains at 15, 3, and 1 km horizontal resolution. - Number of grids: 119 × 119, 115 × 115, 111 × 111		City-scale	[152]
	Mixed forest, combined mixed forest and grasslands, Combined mixed shrublands and grasslands	- Three nested domains at 18, 6, and 2 km horizontal resolution. −38 vertical levels		Regional-scale	[107]
ENVI-met	Vertical/traditional/quality improved greening	- Model domain: 1000 × 910 × 45 m - Grid size: 200 × 182 × 15 - Resolution: 5 × 5 × 3 m		Neighbourhood-scale	[87]
	Green roof, green wall	- Model domain: 126 × 94 × 60 m - Grid size: 63 × 47 × 30 - Resolution: 2 × 2 × 2 m		Street-scale	[141]
	Street tree, green facade, extensive and intensive green roof	- Model domain: 250 × 250 × 200 m - Resolution: 2 × 2 × 2 m		Street-scale	[93]
	Garden	- Model domain: 220 × 150 m - Grid size: 100 × 68 × 30 - Resolution: 2.2 × 2.2 × 2 m (z increased with height after 17 m)		Street-scale	[153]
	Tree canopy	- Model domain: 50 m × variable width - Resolution: 2 × 2 × 2 m		Street-scale	[127]
	Greenway, grove, green roof	- Model domain: 300 × 160 m - Grid size: 150 × 80 × 30 - Resolution: 2 × 2 × 2 m		Street-scale	[111]
	Trees, green roofs, green facades	- Grid size: 87 × 100 × 25 - Resolution: 2 × 2 × 2 m		Street-scale	[154]
UGBE	Urban parks	- Model domain: 236 × 173 × 70 m manually divided into 165 zones - Horizontally grouped in three layers at 0–10 m, 10–20 m, and 20–50 m above the ground		Street-scale	[81]
Hybrid					
Method	GI type	Duration	Description	Scale	Reference
Field measurement/ENVI-met simulation	Green roof, green wall	June to September	- Grid size: 63 × 47 - Resolution: 2m × 2m × 2m	Street-scale	[141]
Field measurement/ENVI-met simulation	Green roofs, green façade, street tree	One day in August	- Grid size: 250 × 250 × 200 - Resolution: 2m × 2m × 2m	Neighbourhood-scale	[93]
Field measurement/ENVI-met simulation	Greenway, green roof	Ten days across different seasons	- Grid size: 300 × 160 - Resolution: 2 m	Neighbourhood-scale	[135]

a Typical instruments used for thermal comfort monitoring include Delta ohm, HOBO, Hanwell, Testo, Kestrel, fisheye images in addition to questionnaire surveys.

b Typical simulation tools used for thermal comfort modelling include ENVI-met, WRF, UGBE, and RayMan model.

c GL: Ground Level.

Many studies aimed to evaluate the thermal comfort improvement benefit provided by various GI types. Cheung and Jim (2018) compared PET and UTCI thermal comfort indices through employing field measurement, RayMan and BioKlima models within an urban park. The comparison of PET and UTCI thermal stress classification in the study revealed some of their intrinsic differences. The UTCI scale is based on human physiological strain, whereas PET is based on human comfort. PET showed a propensity to overestimate heat stress compared to other methods, which was attributed partly to the RayMan model's constant clothing insulation value and partly to the nature of the scale [96].

Wang et al. (2018) collected subjective thermal sensation data through a questionnaire alongside field measurements at three different sites in Guangzhou. The questionnaire consisted of a personal information section including age, gender, nationality, weight, height, length of residency as well as individuals' thermal comfort condition and preferences for changes in temperature, humidity, and wind speed. The multiple-choice thermal conditions questions were evaluated based on a 7-point scale according to ASHRAE Standard 55. The activity level and clothing value were measured by the researcher before or after each questionnaire was completed. The results were analysed to identify the relationship between microclimatic variables and individuals’ thermal comfort perception [68].

The relationship between actual thermal sensation and measured thermal comfort level is an important aspect of understanding thermal conditions. While thermal comfort is typically assessed using standardised models that take into account factors such as air temperature, humidity, clothing insulation, and metabolic rate, these models do not always accurately capture an individual's actual thermal sensation. Individual differences in physiology, psychological factors, and personal preferences can lead to discrepancies between the predicted and the real performance of GI. To create environments that truly meet the occupants' thermal comfort needs, it is useful to incorporate real-time feedback from occupants and their subjective assessments of comfort. This comprehensive approach promotes occupant satisfaction and well-being by ensuring a more user-centric assessment of thermal comfort.

Table 4 provides a summary of some earlier studies using monitoring methods to assess thermal comfort conditions. These monitoring studies were examined at street and neighbourhood-scales over periods varying from one day to four months in one season or on some days during the summer and winter seasons. In order to measure meteorological parameters, researchers carried out field campaigns on representative days, placing instruments at heights of 1.1–2.0 m (Table 4).

Surveys and interviews with between 100 and 150 participants were used to investigate personal characteristics like clothing insulation, gender, and metabolic rate [[140], [146], [155]].

#### 5.2.3 Remote sensing

Remote sensing methods have been used in some of the previous studies to evaluate the effect of GI on thermal comfort conditions. Simple algorithms for the UTCI have been developed and shown to be effective when used with remotely sensed data [156]. Mushore et al. (2023) used multi-spectral Landsat 8 and Landsat 9 data to map LCZs in the eThekwini municipality, South Africa, and LST to adjust and derive thermal comfort conditions based on UTCI on the extremely low- and extremely high-temperature periods [157].

Feng et al. (2020) investigated the impacts of landscape composition on thermal comfort. Twelve image scenes were used including ten Landsat 5/7 TM/ETM + scenes and two successive Landsat 8 OLI/TIRS in three seasons (winter, spring and summer) in 1994, 2000, 2010 and 2013. They used five landscape metrics including the Percentage of Landscape, Aggregation Index, Landscape Division index, patch cohesion index, and Shannon's Diversity Index for defining landscape composition. They applied the Discomfort Index (DI) to measure human discomfort in different landscape settings [158]. This index is a linear equation to quantify outdoor thermal comfort conditions by combining air temperature and relative humidity [150]. It can be effectively applied through remote sensing methods as satellite data provides spatial analysis for thermal information. This index is defined as:

(1) $$DI=T_{a}-0.55\left(1-0.01RH\right)\left(T_{a}-58\right)$$

Where $T_{a}$ is in Fahrenheit and RH is in %. Mutani and Todeschi (2020) also employed DI to evaluate the effect of green roofs on surrounding urban environment thermal comfort conditions in Turin, Italy. They used Landsat 8-OLI/TIRS images to identify the existing and potential green roofs in eight areas on a reference day with a cloud cover of less than 5 % [150]. Stathopoulou et al. (2015) compared the DI values acquired by thermal infrared data from satellite data and DI values obtained from recorded values at standard meteorological stations. Statistical analysis showed a good agreement (r^2^ = 0.79) between satellite-estimated DI and station-obtained DI values [159]. While DI has been employed in previous studies, other indices such as UTCI are more common because of their ability to incorporate a broader range of climatic parameters.

Najafizadeh et al. (2021) employed 65 Landsat-5 and Landsat-8 images to map and monitor thermal comfort in Tehran between 1989 and 2019. The thermal comfort condition was investigated by the Urban Thermal Field Variance Index (UTFVI). The decadal UTFVI maps revealed notable thermal comfort degradation in Tehran, Iran, by which in 2019, ∼52 % of the city was identified as the region with the worst environmental condition [148]. Sharma et al. (2021) also used Landsat-5™ and Landsat-8 images with a resolution of 30 m for the months of April 2011 and 2019 to evaluate thermal comfort conditions in Noida, India, using the UTFVI index. They categorised the UTFVI index values into six classes to analyse temporal variations in thermal comfort conditions, revealing a trend of declining thermal comfort over the study period [149].

The UTFVI indicator, which is derived from the following equation, is commonly used [[143], [148], [149], [160], [161], [162]] to evaluate the quality of the urban environment and urban health by measuring the thermal comfort conditions in the surrounding area.

(2) $$UTFVI=\frac{LST-{LST}_{mean}}{{LST}_{mean}}$$

Remote sensing methods have been used in earlier studies to measure thermal comfort levels at the city and regional levels. For this purpose, in addition to the UTCI thermal comfort index, DI and UTFVI indices have been widely used to derive thermal comfort conditions through satellite images. The UTFVI directly incorporates LST data, whereas the DI depends on air temperature and relative humidity parameters. This makes the UTFVI a useful tool for assessing the improvement of thermal comfort associated with GI via remote sensing. The disadvantage of the remote sensing approach is its limited spatial resolution since thermal comfort conditions are highly reliant on microclimate variations and small-scale features such as trees, waterbodies, buildings, and urban structures. However, remote sensing methods provide a way to evaluate variations in thermal comfort levels over time and investigate the effects of land cover changes.

#### 5.2.4 Modelling methods

Outdoor thermal comfort modelling approaches evaluate weather conditions, urban layout, and human activities to enhance comfort and well-being in open spaces. By employing simulations and data analysis, these methods help urban planners and designers in creating outdoor environments that promote comfort and liveability. For this purpose, various studies employed different modelling tools and thermal comfort indices to evaluate the effectiveness of different GI types in improving thermal comfort conditions.

Imran et al. (2019) used the WRF model with the Advanced Research WRF dynamics solver. They conducted a simulation for four severe heatwave days and employed the UTCI thermal comfort index to evaluate the impacts of GI on outdoor thermal comfort in Melbourne, Australia. They concluded that the vegetated patches made a substantial improvement in human thermal comfort between the evening and early morning, but not during the strong thermal stress of the day [107]. Wang et al. (2022) assessed pedestrians' thermal comfort during a heatwave in Berlin by employing the WRF/UCM model, quantifying the conditions using the UTCI index. The study integrated the WRF model with UCM. The UCM was incorporated in the initial model layer and accounted for canopy processes, including shadowing, reflections, and radiation trapping, while also addressing the surface energy balance of roofs, walls, and roads, along with anthropogenic heat emissions. The results showed that green roofs demonstrated decreased wind speed, decreased mean radiant temperature, and increased relative humidity in addition to lowering temperatures compared to the current state without GI. The overall effect of these modifications led to a decrease in city-scale UTCI [111].

A Heat Health Impact method which is a multi-parameter model applying inter-disciplinary methods based on the UTCI index was developed through simulating a representative day of heatwave to quantify the benefits of urban heat mitigation scenarios on human heat balance and population mortality in Sydney, Australia. In this method, the impact of GI on the human heat balance, UTCI changes under each adaptation strategy, population-weighted mean UTCI across the city, and heat-mortality rate for a typical summer heatwave episode were employed to calculate the UTCI heatwave-attributable mortality change. This study used remote sensing data and data from 10 climate stations to run the WRF model. The results highlighted the effective impact of GI scenarios (adding trees, adding trees combined with green roofs, adding trees combined with green roofs and a 50 % increase of evapotranspiration rate over the planted green areas) on reducing daily average UTCI during the heat wave period at city scale (Sydney), contributing to a reduction in heat-attributable deaths of up to 11.7 per day [151].

**Fig. 4:**
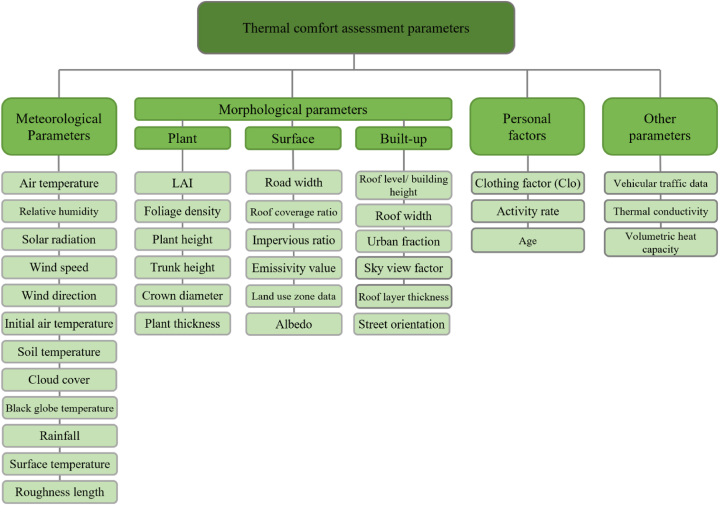
A set of parameters derived from previous papers for monitoring and modelling the thermal comfort benefit provided by GIs and thereby evaluating their performance at various scales.

Zölch et al. (2016) investigated the effect of trees, green roofs, and green facades on pedestrian thermal comfort conditions in high-density residential areas. They employed the ENVI-met simulation tool to analyse the thermal comfort conditions for current and future climate conditions. The results revealed that trees have the highest effect with an average PET reduction of 13 %, followed by green facades (5–10 %); however, the strategic placement of the vegetation in heat-exposed areas is a more prominent factor than the type and the percentage of green cover [154]. Zölch et al. (2019) employed mixed approaches through field measurement and use of the ENVI-met simulation tool to evaluate thermal comfort conditions in Munich public squares to find out the effects of GI on improving thermal comfort. The meteorological data was extracted from weather stations for a typical hot summer day. The soil temperature was measured underneath the canopy of selected trees using Tensiomark sensors as the initial soil temperature is one of the required inputs for ENVI-met simulation (Fig. 4) to capture the complex interactions between ground and atmosphere that affect outdoor thermal comfort. The results from simulations which were run for 48h confirmed that GI reduces the daytime PET, while the tree crowns trap and hinder the hot air contributing to an increase in nighttime PET [163]. The metric from a study conducted in Guangzhou employing the PET index revealed that street trees were the best GI strategy among the monitored scenarios through the ENVI-met simulation tool, whereas adding green roofs showed minimal improvements in pedestrians’ thermal comfort. The model utilised meteorological records from the meteorological station and was simulated for 24 h with a 1-h interval [58]. Morakinyo et al. (2020) involved a combination of various generic tree forms and different characteristic urban morphology to identify the importance of these parameters on the thermal situation and consequently the thermal comfort conditions using the ENVI-met model. They recognized that informed tree species selection in the respective urban canyon can reduce mean values of PET compared with uninformed tree species selection scenarios [127]. ENVI-met uses BIO-met as a post-processing tool to calculate human thermal comfort based on simulation data. It provides an assessment of dynamic thermal Comfort and a range of static indices such as PET and UTCI (ENVI-met Applications Homepage, 2023). Zhang et al. (2022) compared the BIO-met output with experimental data to verify the accuracy and reliability of BIO-met results. The relationship between the software-calculated PET and UTCI and the perceived thermal comfort conditions was derived from a questionnaire survey with 30 participants. The results revealed that despite the slight deviation between simulation results and actual conditions, there were cross-values between two sets of data [164]. ENVI-met simulation tool has a high spatial resolution that considers physiological vegetation processes and the inclusion of a plant database within the model provides the opportunity to incorporate vegetation profiles.

In the reviewed studies on thermal comfort assessment using the WRF simulation tools, RayMan software was used to quantify the impact of temperature changes under the GI interventions on the UTCI thermal comfort index. The following variables from the WRF modelling were input into the RayMan model: date, time, longitude, latitude, air temperature, relative humidity, wind speed, land surface temperature, albedo, and global radiation [111,151]. The WRF simulations were conducted on city- and neighbourhood-scales offering spatial coverage of thermal comfort conditions in urban areas and regions while the fine-scale details of local features, such as the effects of individual buildings or variations in land cover, which are important for assessing thermal comfort in urban areas are not considered. ENVI-met is a street-scale simulation tool which can also be used at small neighbourhood scales. In the reviewed studies the resolution of the grids varied from 2 to 5 m which can cover the fine-scale details of the urban environments (Table 4).

The thermal comfort conditions do not only depend on meteorological and physical parameters but also some studies highlighted the effects of individuals' expectations on their thermal comfort conditions [165,166]. The benefit of GI in terms of actual thermal comfort condition is higher than the measured improvement of thermal comfort indices as it provides individuals with a thermally comfortable environment expectation. Therefore, combining monitoring and questionnaire surveys for evaluating outdoor thermal comfort provides a comprehensive understanding by incorporating both objective and subjective perspectives. This strategy can be enhanced by adding a modelling method to provide a holistic understanding of outdoor thermal comfort conditions. This integrated approach validates and calibrates measurements, enables comprehensive scenario evaluations, and provides real-time and long-term data as well as facilitating stakeholder engagement and collaboration. Monitoring data provides objective evidence, modelling offers visual representations of different scenarios, and questionnaire surveys capture individuals’ actual thermal preferences. These outputs can be used to engage stakeholders, such as urban planners, architects, policymakers, and the public, in discussions and decision-making processes related to outdoor thermal comfort.

The reviewed studies employed monitoring, remote sensing, and modelling methods to evaluate the thermal comfort benefits offered by GIs. The parameters considered in these studies are grouped into four categories: 1) Meteorological parameters, 2) Morphological parameters encompassing plant, surface, and built-up area characteristics, 3) personal factors, and 4) other parameters (Fig. 4). These parameters are measurable through field measurements, stationary databases, field surveys, satellite data, and other sources. They serve as essential inputs for simulation tools, aiding in the estimation of the thermal comfort benefits derived from GIs.

### 5.3 Air quality

#### 5.3.1 Indicators

Air quality indicators offer important details on the concentration of different pollutants and their potential effects on the surrounding environment and human health. The main pollutants are categorised into: 1) PM including fine and coarse particulate matter (PM_2.5_ and PM_10_), and 2) gaseous pollutants including NO_2_, O_3_, SO_2_, CO, and VOCs. The overall AQI is an indicator that combines data on various pollutants to provide a value representing overall air quality.

#### 5.3.2 Monitoring methods

The effectiveness of GI at the local or street scale can be measured by applying a range of methods, including: (1) monitoring pollutant concentrations at street level near GI (e.g., both sides of a vegetation barrier) to evaluate the combined impacts of dispersion and deposition; (2) utilising microscopy imaging techniques like optical microscopy, confocal laser scanning microscopy, scanning electron microscopy (SEM) to analyse pollutant deposition; (3) employing gravimetric procedures or laser granularity instruments to quantitatively measure pollutant deposition and assess its mass; and (4) supplementing deposition assessments by evaluating the elemental composition of deposited pollutants [22].

In order to measure the improvement in air quality provided by GI on a local scale, in-situ measurement has been widely adopted in previous studies to analyse the pollutant concentrations at different points to explore concentration changes provided by GI. A wide range of tools has been employed varying from low-cost sensors to other tools (Table 5). In addition to the field measurements, some studies recorded traffic volume using video cameras during the sampling to estimate real-time traffic emissions [82].

**Table 5:** Summary of previous studies and derived information on air pollution monitoring (instruments, temporal period, measurement height, and scale) and modelling studies (simulation tools, domain size, grid size, resolution, and scale).

Monitoring^a^					
Instruments	GI type	Duration of measurement	Measurement height	Scale	Reference
AQ Mesh V5.0	Green barrier	April to October	1.7 m above GL^c^	Street-scale	[84]
Alphasense CO-B4, Alphasense NO_2_-B43F, Plantower PMS 5003	Hedges	February to June	1.5–1.7 m above GL	Street-scale	[75]
GRIMM (EDM 107 and 11-C), P-TRAK 8525, MicroAeth AE51	Hedges, trees, mix of trees and hedges, shrubs	Five days per each site from 8:00 to 18:00 (30 days in total)	1.5 m above GL	Street-scale	[80]
Tecan CLD 700AL, PUF disk sampler Klaus Ziemer GmbH, passive diffusion samplers	Woodland, park, trees	March, April, July, August, and September	2.5 m above GL	Street- and neighbourhood-scale	[167]
TSI DustTrak II	Hedges, green wall	One day(August) - 14:00 to 16:00	1.4 m above GL	Steet-scale	[82]
MicroAeth AE51, DiSCmini	School greenery	Two one-week campaigns in warm and cold season - 8 h per day	0.7–1.5 m above GL	Street-scale	[168]
42C Monitor Labs 9841A, TEi-49C, TEOM 1400AB, Drager-Pac III	Urban park, urban square, street canyon	Eight days (June and January)	–	Street- and neighbourhood-scale	[169]
Ogawa passive sampler		January, April, July, and October	2 m above GL	Neighbourhood-scale	[170]
Remote sensing					
Satellite	GI type	Duration	Description	Scale	Reference
Sentinel-5P and Landsat 8	Urban greenery	Summer and winter from 2019 to 2021	Resolution of 1 km	City-scale	[171]
Worldview-3	Trees	One day (August 2017)	Panchromatic band at 30 cm resolution	City-scale	[172]
Sentinel-2	Forests	Year 2019	Resolution of 10 m	City-scale	[173]
MODIS	Urban greenery	January to December 2016	Resolution of 1 km	City-scale	[174]
Modelling^b^					
Simulation tool	GI type	Model description		Scale	Reference
ENVI-met	Green wall, green roof	- Model domain: 120 × 120 × 30 m and 274 × 274 × 50 m - Resolution: 2 × 2 × 2 m and 3 × 3 × 3 m		Street-scale	[175]
	Hedge	- Domain: 50 × 30 × 40 m - Resolution: 0.5 × 0.5 × 0.4–2 m		Street-scale	[176]
	Hedge, green wall	- Domain: 160 × 40 m - Resolution: 1 × 1 × 1 m		Street-scale	[82]
openFOAM	Trees	- The mesh was made of about 4 million hexahedral cells. - Resolution: 1.25 × 1.25 × 0.5 m		Street-scale	[177]
	Trees, grass	- Domain: 2 × 2 km - Resolution: 1 m for building, 2 m for trees, 4 m for grass		City-scale	[178]
i-Tree Eco	Trees, green wall, green roof	- Grid size: 200 × 200 m −88 plots to collect field data each plot had an area of 0.1 ha		Neighbourhood-scale	[179]
EMEP4UK	Woodland, grassland	- Resolution: 5 × 5 km - Buffer size varied between 5 m and 500 m		Regional-scale	[70]
	Forests	- Resolution: 4 and 20 km		City-scale	[83]
VADIS	Green roof, trees	- Domain: 753 × 753 × 126 m - Resolution: 3 × 3 × 3 m		Neighbourhood-scale	[180]
	Trees	- Domain: 700 × 650 m and 800 × 800 m - Resolution: up to 1000 m^3^		Neighbourhood-scale	[181]
Hybrid					
Method	GI type	Duration	Description	Scale	Reference
Field measurement/ENVI-met simulation	Hedge, green wall	One day in August	Domain size: 50m × 30m - Resolution: 0.5m × 0.5m × 0.4m	Street-scale	[176]

a Typical instruments used for air quality field measurement include DMS, TSI DustTrak, microAeth, GRIMM, P-Trak, AQ Mesh, diffusion tubes, Aeroqual, and PMS.

b Typical simulation tools used for air quality modelling ENVI-met, OpenFOAM, i-Tree Eco, VADIS, and CMAQ, EMEP, and other CFD tools.

c GL: Ground Level.

Microscopy imaging techniques are another widely used method to quantify particle deposition on leaves. Among different imaging techniques, SEM/ESEM is the most used method. Using ESEM and image analysis tools, researchers have determined the particle number concentrations for various PM fractions per mm^2^ by counting particles deposited on leaves [182,183].

Another method to determine the potential of various GIs to remove pollutants is measuring deposited PM through washing the leaf and performing a gravimetric analysis of the removed pollutants [184]. Different techniques have been employed to analyse the elemental composition of particles deposited on leaves and to explore compositional changes in the presence of vegetation including SEM equipped with energy-dispersive X-ray spectroscopy (SEM-EDS/EDX/EDAX) and inductively coupled plasma optical emission spectrometry (ICP-OES) [[184], [185], [186]].

A summary of some previous studies that evaluated the benefits of various GI types on air pollution using monitoring techniques is given in Table 5. These monitoring studies were conducted on certain days in the summer and winter or over periods ranging from one day to seven months at street and neighbourhood-scales. Researchers conducted field campaigns on representative days to measure the concentrations of pollutants, positioning instruments at heights ranging from 0.7 to 2.5m (Table 5).

#### 5.3.3 Remote sensing

To monitor the air quality on a larger scale remote sensing methods can be adopted. Many studies used satellite data to assess air pollution concentrations at city- and regional-scale. Bakaeva and Le (2022) used Landsat 8 OLI to develop a model for determining fine dust PM_2.5_ in Moscow. They pre-processed the images by converting numeric values into a spectral wavelength or spectral reflectivity with several levels of radiation correction. Through this process, it is essential to eliminate the influence of atmospheric conditions on image quality by carrying out an atmospheric correction [187]. Rahman et al. (2023) used Sentinel-5P satellite data to derive NO_2_ concentrations alongside Landsat 8 for calculating NDVI and enhanced vegetation index values. They divided the research regions (Delhi and Dhaka, India) into a 1 × 1 sq.km grid to carry out statistical analysis. They also investigated the relationship between NO_2_ concentration and mean LST, finding a positive correlation between these values [171].

Basharat et al. (2023) utilised Aqua-MODIS and Angstrom Exponent (AE) data to analyse the spatiotemporal seasonal variability over Pakistan from 2002 to 2021. They collected data on Aerosol Optical Depth (AOD) and AE to track their seasonal variations and spatial variability across all Pakistani provinces [188]. The AOD represents the way aerosol particles in the atmosphere attenuate sunlight. Particulate matter concentrations are higher when the AOD is high. A spatial distribution of aerosol loading is provided by this method [188,189]. The AE is a parameter used in atmospheric science that provides information about the size of aerosol particles by utilising AOD. It is used to characterise the wavelength dependence of aerosol optical properties. Specifically, the AE is applied to describe the relationship between aerosol optical thickness at different wavelengths, often in the visible and near-infrared spectral range [[188], [189], [190]]. The results of this study showed a negative correlation between the aerosol concentrations and NDVI values during different seasons.

Some other studies utilise satellite data solely for calculating GI conditions and obtain data related to pollutant levels from monitoring stations. By examining these data along with NDVI, they assess the impact of vegetation on pollutant concentrations. Araminiene et al. (2023) used Worldview-3 satellite imagery to investigate the effect of trees on air pollution over a large study area (56 km^2^) in the city of Kaunas, Lithuania [172]. Muresan et al. (2022) employed a multiscale approach including satellite data and monitoring to quantify the role of the urban and peri-urban forests in removing O_3_ and PM_10_ concentrations from the Municipality of Ferrara. They retrieved monthly PM_10_ and O_3_ concentrations from three monitoring stations and used Sentinel-2 Level-2A satellite data to classify land use/land cover and to compute the seasonal LAI over the study area. They calculated the seasonal LAI using the biophysical processor module of the Sentinel Application Platform software [173].

Bechle et al. (2013) compared NO_2_ concentrations obtained from satellite data to ground-level NO_2_ concentrations within a large urban area by relating satellite column measurements to ground-level concentrations. Through comparing 4138 sets of paired data, they found out that although there are more data gaps in satellite data than the ground monitors (due to cloud contamination and imposed limits on pixel size) the spatial correlation between satellite data and in-situ measurements is strong (*r* = 0.93 for annual average data) [191].

Remote sensing methods have significant advantages in assessing the improvement in air pollution offered by GIs. Their large-scale coverage, continuous monitoring capabilities, and multispectral data all help to provide a comprehensive understanding of regional air quality dynamics. In the reviewed studies in which this method was employed, the scale of the investigation area encompassed a range of scales, extending from the city-scale [188,172,173] to larger geographic extents, including regional [171] and the entire country [188]. However, limitations include a lack of detailed vertical resolution, sensitivity to atmospheric conditions, difficulties distinguishing specific sources, and the complexities of capturing the nuanced effects of GI, coupled with the relatively coarse resolution of the available data.

#### 5.3.4 Modelling methods

The modelling approach to analyse air pollution in an urban context has been widely used in previous studies. These approaches are complemented by monitoring methods to validate and calibrate the models to ensure accurate results. Modelling methods can provide scenario analysis, forecasting, source apportionment, temporal resolution, and spatial coverage.

Some studies employed dry deposition models to quantify the pollution reduction potential of GIs such as the Urban Forest Effects (UFORE) model. UFORE provides a method for quantifying the structure, deposition of air pollutants, emission of BVOCs, carbon storage, and annual carbon sequestration of urban vegetation, as well as identifying the species that are most effective at improving local air quality [192]. Currie and Bass (2008) conducted a study to assess the effects of different GI scenarios (green walls, trees, shrubs, and grass) on air pollution in a neighbourhood-scale study area using the UFORE model [193]. As an improved version of UFORE that can also assess the monetary values of GI's environmental services, i-Tree Eco was introduced. This model was first applied to several American cities to evaluate the improvement of air quality and the many ecosystem services provided by GI, including carbon storage, carbon sequestration, and energy savings [194,195]. This model can be applied at various scales, based on previous studies the model was applied on a neighbourhood-scale [179], city-scale [196], and a regional-scale [197]. Jayasooriya et al. (2017) used the local meteorological and air quality data provided in the i-tree Eco database alongside the surface weather data provided by weather stations to assess the pollutant removal of different GI scenarios including trees, green walls, and green roofs [179]. Yao et al. (2022) adopted the i-Tree Eco model to explore optimal GI by comparing the air pollutants removal provided by adding different types and amounts of greenery. They used the LCZ to propose a practical approach that combines the LCZ concept with the i-Tree Eco simulation to improve air quality [197]. Villani et al. (2021) employed a PMSS (Parallel Micro-SWIFT-micro-SPRAY) microscale model to simulate the air quality of an innovative wall-type GI. This model can be used for local scale and microscale simulations but cannot calculate the airflow through a porous medium, such as a tree, because the porosity of obstacles has not been implemented [198]. Other models such as the CHIMERE air quality model [199] exhibit minor variations in comparison to the parameterizations utilised by i-Tree [196]. The CHIMERE chemical transport model was first introduced as a box model covering the Paris area and then the geographical domain was extended over European countries [200]. This model has been used in studies focusing on ozone and PM_10_ from the city-scale [201] to continental-scales [202].

Some studies employed the European Monitoring and Evaluation Programme (EMEP) for transboundary large-scale transported air pollutants. The model can be applied on scales ranging from local-scale (1 km grid size) to global-scale (with 1-degree resolution) due to the flexible processing of chemical schemes, meteorological parameters, and nesting capability [203]. Nemitz et al. (2020) used the EMEP4UK model to quantify the effect of large-scale urban vegetation on air pollution. They used seven existing land cover classes including deciduous forest, coniferous forest, crops, semi-natural land, water, and urban of the EMEP model as well as the WRF model for the meteorological inputs for their analysis. Using this model the effects of different urban tree-planting scenarios were assessed for total PM_2.5_, SO_2_, NO_2_, O_3_, and NH_3_ [79]. Jones et al. (2019) estimated health benefits from the change in pollutant concentrations by GIs using EMEP4UK with the same model set-up. They applied a variable-sized spatial buffer to map and quantify urban GI, and to estimate the health benefits from improvements to air quality provided by existing GI (urban grassland, trees and waterbodies) for the year 2015 [70]. Meta-models to quantify the health benefits of air-pollution removal by trees, derived from bespoke EMEP4UK scenarios, have been used to assess potential interventions of tree-planting and agricultural land use change at national scale in Wales, UK, to directly inform government policy [204].The Community Multiscale Air Quality Modeling System is developed by the United States Environmental Protection Agency and can be used at various spatial scales ranging from city-scale to regional and global-scales [205]. To simulate the impact of vegetation increase on air quality, Zhang et al. (2020) used WRF and CMAQ models on a domain coverage of a 12-km horizontal resolution and a 4-km horizontal resolution for the inner domain with 35 vertical layers for both the base scenario and GI land use scenarios using two land surface models. Their findings emphasised a region-specific non-linear process feedback from GI on regional air quality, as well as the importance of complete coupled meteorological-air quality modelling systems and an accurate land surface model for evaluating these consequences [206].

Rafael et al. (2018) evaluated the influence of a set of resilience measures by employing WRF and CFD model VADIS (pollutant dispersion in the atmosphere under variable wind conditions) at a neighbourhood-scale [180]. VADIS is a tool developed for estimating pollutant dispersion in complex urban areas from traffic emissions that identifies local hotspots and supports multi-obstacles, sources, and variable flow and emissions, assessing short-term concentrations in urban morphologies [181]. The WRF model was used to initialise the CFD model, and the Noah land surface scheme was combined with a single-layer urban UCM to better represent the physical processes involved in an urban environment (exchange of heat, momentum, and water vapour) [180].

Another simulation tool which has been widely used in previous studies is ENVI-met. ENVI-met is a three-dimensional computational fluid dynamics and micro-climate model [207] with a flow solver based on the Reynolds Averaged Navier-Stokes (RANS) equations. Morakinyo and Lam (2016) analysed the impact of a near-road vegetation barrier on air quality using the ENVI-met integrated dispersion-deposition simulation tool. A method called "distance to maximum concentration" has been proposed to determine the ideal position from the source and thickness of the vegetation barrier for improved dispersion and deposition-based benefit, respectively. This method is based on the distance between the source and the point of peak concentration before the dwindling concentration downwind begins [176]. To investigate the effect of hedges and green walls and dimensions on the near-road air quality Morakinyo et al. employed an integrated dispersion-deposition approach using ENVI-met simulation and field measurement [82].

While ENVI-met has been widely used in many previous papers, Sun et al. (2021) compared the applicability of ENVI-met, CAL3QHC, and ANSYS Fluent for modelling pollutant dispersion at a road intersection focusing on PM_2.5_ as the subject matter. They developed a dynamic emission factor model based on the Cell Transmission Model and the Portable Emission Measurement System experiment. The results of this study showed that although ENVI-met excels in evaluating the correlation between PM concentrations and intersecting meteorological factors, ANSYS Fluent is a better simulation tool for predicting PM_2.5_ concentrations. CAL3QHC and ANSYS Fluent simulation results were within acceptable values [208].

In order to facilitate efficient GI design and management for air pollution abatement in street canyons, Barwise et al. (2021) co-developed a public engagement and decision support tool namely HedgeDATE (Hedge Design for Abatement of Traffic Emission) using user-directional input data to provide recommendations such as species plans and projections for reducing pollution [209].

Hashad et al. (2021) predicted size-resolved and location-dependent particle concentrations downwind of different vegetation barrier designs by training machine learning models using data from a wide range of CFD simulations. They investigated five ML techniques, including linear regression, support vector machine, random forest, XGBoost, and neural networks. To accurately capture its complexity and improve the overall accuracy, downwind region-specific models were created. The feature space was built using variables such as vegetation width, height, LAI, particle size, leaf area density, and wind speed at various heights [210].

**Fig. 5:**
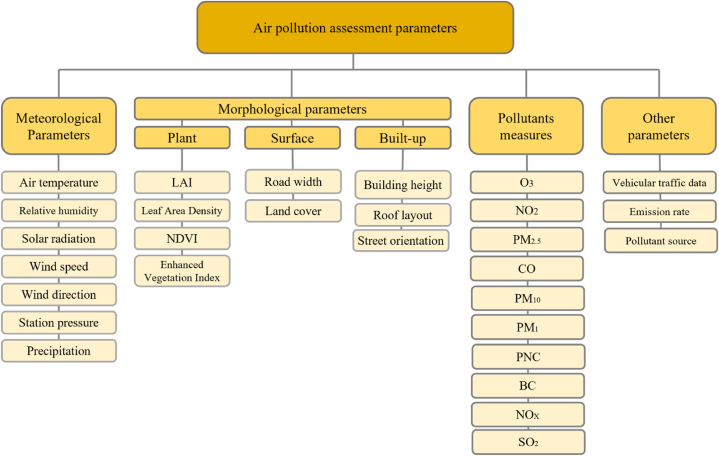
A set of parameters derived from previous papers for monitoring and modelling the air pollution improvement benefit provided by GIs and thereby evaluating their performance at various scales.

The reviewed studies used monitoring, remote sensing, and modelling to determine how GI improves air quality. The factors examined in these studies are categorised into meteorological factors, morphological parameters, measured pollutants, and other parameters (Fig. 5). These parameters, measured using various methods, serve as critical inputs for simulation tools that estimate pollutant removal by GIs. Studies have confirmed that the effect of GI on air quality is complex, scale- and site-specific. For deposition models, outcomes on particulate matter concentrations are largely a function of pollutant concentrations and tree-cover. By contrast, the geometry of the built environment dominates the nature of vegetation effects for dispersion at fine scale. For instance, some studies showed that in open road environments, a mixture of trees and bushes can act as barriers to improving air quality behind them [[211], [212], [213]] if the GI is not arranged properly, it may lead to a decline in air quality. Moreover, previous studies revealed that seasonal variations in the street canyon's pollutant exposure were caused by various tree species. Pollutants were trapped in street canyons with deciduous trees during the summer and higher pollutant concentrations were discovered in street canyons with evergreen trees during the winter [212,214]). Therefore, it is necessary to analyse the air quality in various urban areas comprehensively by taking into consideration all the influential parameters. For this purpose, it is vital that the investigation methods should be carefully selected considering the limitations of each method and the complexity of the GI performance in different urban contexts.

### 5.4 Limitations and advantages of monitoring, remote sensing, and modelling methods

Various approaches for assessing the benefits of GI focusing on heat mitigation, thermal comfort, and air quality have been discussed in this paper. Different monitoring, modelling, and remote sensing methods have been employed in the reviewed studies. Each of these methods has some strengths and limitations which need to be taken into account to realise the real performance and efficacy of different GI types.

Monitoring methods can offer real-time data providing direct and accurate information on the particular environmental conditions as well as offering detailed insights into specific parameters while utilising different monitoring devices and sensors. However, the lack of spatial coverage may result in data gaps resulting in challenges in a comprehensive assessment of GI benefits. Moreover, the high equipment costs, maintenance, and calibration requirements make the monitoring resource intensive.

Remote sensing methods provide broad-scale coverage which makes it a suitable tool for assessing large urban areas and the time-series data can reveal trends and changes over time. However, the remote sensing data may lack the required resolution to capture the localised effects limiting their applicability for micro-scale assessments.

Modelling methods enable the simulation of complex environmental processes and can project future scenarios, helping urban planners to anticipate the long-term impacts of GI implementation. Conversely, they rely heavily on input data and assumptions which can introduce some uncertainties and may cause different results compared to real-time conditions.

Among various modelling tools which have been discussed in Sections 5.1.4, 5.2.4, 5.3.4, ENVI-met is widely used for evaluating heat mitigation, thermal comfort, and air quality ecosystem services provided by GI, though it has certain advantages and limitations. In heat mitigation, it models microclimates to evaluate the effectiveness of GIs in reducing surface and air temperatures within the urban environment, but its high computational demands limit broader applications. For outdoor thermal comfort, ENVI-met assesses indices like PET and UTCI at fine spatial resolutions, however representing the individual thermal perceptions remains a limitation. In air pollution reduction, the model simulates pollutant dispersion near GI, providing insights into GI's role in enhancing air quality, but its limited representation of atmospheric chemistry processes can affect accuracy.

**Fig. 6:**
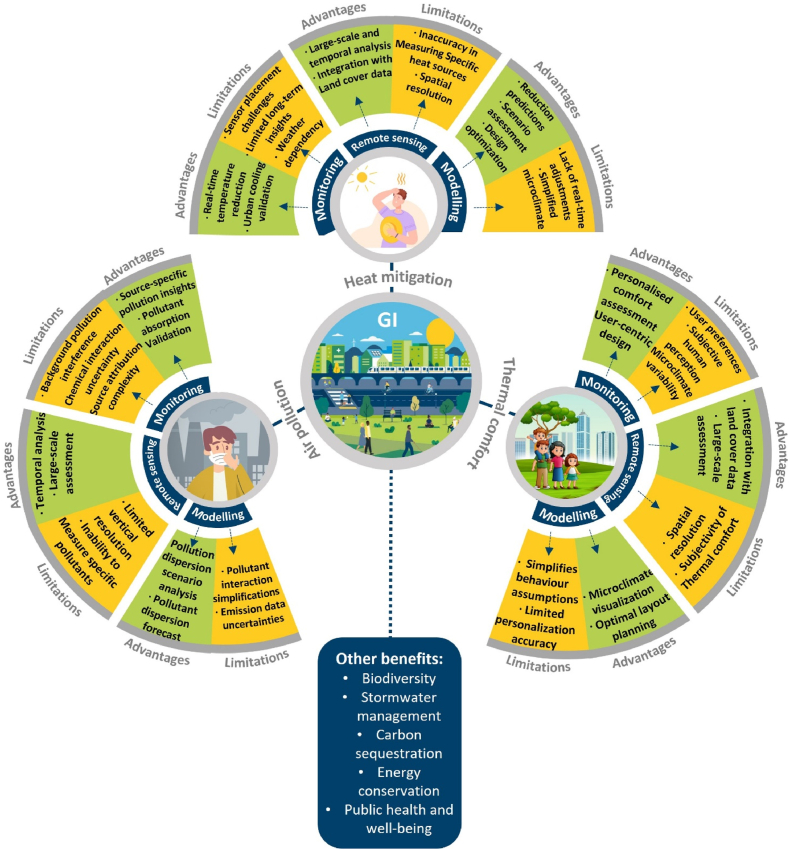
Monitoring, remote sensing, and modelling methods advantages and limitations in terms of heat mitigation, thermal comfort, and air pollution benefits.

Fig. 6 depicts the advantages and limitations of monitoring and modelling methods in assessing the benefits of GI in terms of heat mitigation, thermal comfort, and air pollution. The integration of monitoring, remote sensing, and modelling methods provides a comprehensive approach to assess GI benefits. At local-scales, high-resolution monitoring can provide detailed insights into real-time impacts of GI implementation, with temporal resolution ranging from hourly to monthly. This data is invaluable for calibrating and validating remote sensing data and modelling analysis, thereby enhancing the accuracy of large-scale assessments. At regional scales, remote sensing can bridge the gap in local monitoring to provide broader GI assessments. Satellite or aerial imagery enables the analysis of changes in vegetation cover, urban heat islands, and waterbodies over time including monthly and annual intervals. Combining this data with local monitoring can enhance the temporal and spatial resolution of GI benefits as well as providing real-time data. Modelling extends the results by predicting future evaluation of GI benefits. By incorporating real-time data obtained from monitoring, spatial analysis from remote sensing overtime and modelling, long-term trends and potential impact of different GI types can be evaluated. This integrated approach is useful for assessing the benefits and resilience of GI across a spectrum of time scales, from short-term (hourly) impacts to long-term (decadal) changes. It is important to select a method which aligns with the specific objectives of the analysis, available sources, and the geographical and temporal scales of interest. A holistic understanding of strengths and limitations of each method is crucial for researchers, urban planners, and policymakers aiming to make informed decisions to maximise the advantages of GIs within urban settings.

## 6 Conclusions and recommendations

We summarised and evaluated the existing studies on the co-benefits of GIs in terms of heat mitigation, thermal comfort, and air pollution. The aim was to provide insights into the various methods of assessing the co-benefits of GI and their applicability among various scales, applied procedures, spatial resolution as well as the strengths and limitations. Key conclusions are summarised as follows.

● A variety of indicators have been widely used to evaluate the GI benefits. Air temperature and LST are common indicators used to assess the heat mitigation benefit of GIs. Thermal comfort studies use PET and UTCI as indicators among different methods, but for remote sensing methods, the UTFVI index which allows the evaluation of thermal comfort conditions using LST values has been widely used. Particulate matter (PM_2.5_ and PM_10_) and different gaseous pollutants (NO_2_, O_3_, SO_2_, CO, and VOCs) are taken into account as air quality indicators as well as AQI which combines data from various pollutants.
● The influential parameters that have been used in previous studies have been categorised into: 1) meteorological parameters that can be collected through field measurements, weather stations, satellite data, or other databases; 2) morphological parameters which represent plants, surfaces, and built-up area characteristics (collected from surveys, satellite images, simulation tools databases, and other sources); 3) specified parameters based on research objectives such as ‘landscape metrics’, ‘personal factors’ and ‘pollutants concentrations’ which depends on the studied benefit and objectives; and other parameters. Among the parameters, LAI and some meteorological parameters including air temperature, relative humidity, solar radiation, wind speed, and direction are commonly utilised in assessing all three benefits of GIs.
● The scale of assessment plays a vital role in selecting the appropriate method for evaluating the GI benefits. This review showed that the assessment scale of studies in all three benefits (heat mitigation, thermal comfort, and air quality) relied on the employed methodology. Among the reviewed studies, the scale of monitoring studies was on street and neighbourhood-scale, while the remote sensing method was used for city and regional-scale. The modelling studies encompassed a wide range of scales varying from street-scale to regional-scale depending on the simulation tool specifications, required resolution, grid size limits, and objectives. Based on the reviewed studies, for studies on the street-scale, ENVI-met, openFOAM, ADMS, and UGBE simulation tools have been widely used. The i-Tree Eco, ENVI-met, and ADMS for neighbour-hood scale, openFOAM, EMEP, WRF, MUKLIMO, and ADMS for city-scale studies. Moreover, for regional-scale studies covering large areas WRF and EMEP models have been used.
● Monitoring studies employed various tools for field measurements. These tools were installed at different heights varying from 0.15 to 1.7 m for heat mitigation studies, from 1.1 to 2.0 m for thermal comfort studies, and from 0.7 to 2.5 m above ground level for air pollution studies. Most of the studies consider the average adult height (1.5–1.7 m) or children's height (0.7 m), while some of them consider the centre of gravity for an adult (1.1–1.2 m). Some monitoring studies on heat mitigation provided by green roofs placed the sensors from 0.15 m above the ground to higher levels to evaluate the cooling benefit gradient. In terms of duration of measurements, the monitoring studies ran in different periods ranging from one day to seven months covering different seasons.
● Modelling studies employed a wide range of simulation tools to evaluate the benefits of GIs through simulating different scenarios. Among the reviewed studies, the ADMS and MUKLIMO models have been used for studies on heat mitigation benefits, RayMan and UBGE for thermal comfort studies, and OpenFOAM, i-Tree, EMEP, and VADIS for air quality studies. ENVI-met has been widely used for all three studied benefits with grid resolution ranging from 0.4 to 6 m, while the WRF model has been used for heat mitigation and thermal comfort studies on larger scale studies with a varied resolution of 0.33–18 km. The resolution of the reviewed modelling studies covered a wide range from 0.4m to 20 km which depends on the scale, objectives, tools, and context of the assessment.

The above findings allow us to make the following key recommendations.

● Standardising the use of indicators in assessing GI benefits is crucial for enhancing the reliability and comparability of research findings. By prioritising widely accepted indicators researchers can establish a common ground for evaluating the effectiveness of different GI types. This consistency will not only streamline the analysis of diverse studies but also contribute to the development of a more robust and universally applicable framework for assessing GI benefits.
● A holistic approach to data collection is imperative for studying GI benefits. Incorporating meteorological parameters gathered through field measurements, weather stations, and satellite data ensures a comprehensive understanding of the environmental context. Additionally, including morphological parameters derived from surveys, satellite images, and other investigations contributes valuable insights into the role of plant characteristics, surfaces, and built-up areas. It is crucial to note that, in certain built-up areas, the implementation of GI may lead to adverse effects on air pollution improvement, underscoring the need for precise methods for collecting morphological parameters. Moreover, specifying parameters based on research objectives, such as 'landscape metrics' and 'personal factors' enables a nuanced exploration of GI benefits.
● The assessment scale plays a pivotal role in determining the methodology for evaluating GI benefits. Street and neighbourhood scales are often suitable for monitoring studies, while remote sensing methods are better suited for city and regional scales. Modelling studies span a wide range of scales, from street-scale to regional-scale, contingent upon simulation tool specifications, resolution requirements, grid size limits, and study objectives. Since different methods offer diverse spatial and temporal scales and have some strengths and limitations, it is crucial to select the method that aligns with the specific goals, context and available data sources to tailor assessment methods to the local context to ensure relevance and accuracy.

This review investigated the methodologies for assessing the GI benefits in terms of heat mitigation, thermal comfort, and air pollution. The knowledge gap in the assessment methods provided by GI lies in the need for a more integrated approach that encompasses the diverse benefits of GI across various spatial scales and environmental contexts. Current methodologies often address these aspects separately, resulting in a fragmented understanding of their interconnected effects. Additionally, there is a lack of consensus on the most effective indicators for thermal comfort, requiring further exploration into their applicability and sensitivity across diverse urban settings. Establishing consistent and universally applicable indicators would enhance the comparability and reliability of results. Furthermore, research on the scale-dependent effectiveness of different GI types is limited. There is a need for more comprehensive evaluations that explore how GI performs across varying spatial scales, from street-level to regional. Additionally, nuanced research into the specific contributions of different vegetation configurations and types to the reduction of different air pollutants is required. Bridging these gaps will improve the precision of assessment methodologies, providing valuable insights for more effective and context-specific GI implementation in urban settings.

## Data availability statement

No data was used for the research described in the article.

## CRediT authorship contribution statement

**Soheila Khalili:** Writing – review & editing, Writing – original draft, Visualization, Validation, Methodology, Formal analysis, Conceptualization. **Prashant Kumar:** Writing – review & editing, Visualization, Supervision, Resources, Project administration, Methodology, Funding acquisition, Conceptualization. **Laurence Jones:** Writing – review & editing, Supervision, Project administration, Funding acquisition, Conceptualization.

## Declaration of competing interest

The authors declare that they have no known competing financial interests or personal relationships that could have appeared to influence the work reported in this paper.
