# Supplementary material for: Evaluating the benefits of urban green infrastructure: Methods, indicators, and gaps
Source: Heliyon. 2024 Sep 25;10(19):e38446. doi: 10.1016/j.heliyon.2024.e38446 (PMC11489314; doi:10.1016/j.heliyon.2024.e38446)
Supplement: Multimedia component 1 [file mmc1.docx]

**Supplementary Information (SI)**

For

**Evaluating the benefits of urban green infrastructure: methods, indicators, and gaps**

Soheila Khalili^a^, Prashant Kumar^a,b,*^, Laurence Jones^c,d^

^a^*Global Centre for Clean Air Research (GCARE), Department of Civil and Environmental Engineering, Faculty of Engineering and Physical Sciences, University of Surrey, Guildford GU2 7XH, United Kingdom*

^b^*Institute for Sustainability,* *University of Surrey, Guildford GU2 7XH, Surrey, United Kingdom*

*^c^UK Centre for Ecology & Hydrology, Environment Centre Wales, Deiniol Road, Bangor LL57 2UW, United Kingdom*

*^d^Liverpool Hope University, Department of Geography and Environmental Science, Hope Park, Liverpool L16 9JD, United Kingdom*

*Corresponding author. Address: As above. Email: [p.kumar@surrey.ac.uk](mailto:p.kumar@surrey.ac.uk); [prashant.kumar@cantab.net](mailto:prashant.kumar@cantab.net)

**Table S1.** TSV models based on the meteorological parameters and the corresponding R-values in different Köppen climate types

| **Location** | **Climate type*** | **TSV model**** | **R-value** | **Reference (Year)** |
| --- | --- | --- | --- | --- |
| Marrakech, Morocco | BSh | ASV=0.11Tg-0.71v-0.02RH | - | [1] |
| Phoenix, USA | BWh | ASV=0.30Tg-0.89v+0.07RH | - |  |
| Shanghai, China | Cfa | TSV=0.067T_a_+0.021RH-0.068v+0.008G-2.276 | 0.82 | [2] |
| Guangzhou, China | Cfa | ASV=0.245T_a_-0.457V+0.059T_mrt_+0.013RH-8.527 | 0.60 | [3] |
| Argentina | BWh/BWk | TSV=-0.9796+0.0621T_a_-0.3257v+0.0079HR |  | [4] |
| Wuhan, China | Cfa | TSV=0.0643T_a_+0.00076G-0.16v-0.00376RH-1.382 | 0.67 | [5] |
| Hong Kong | Cwa | TSV=0.1185T_a_-0.6019WS+0.0025SR+0.1155HR-4.77 | 0.91 | [6] |
| Tianjin, China | Bsk, Dwa | TSV=0.0418T_a_+0.0021G+0.28v+0.007RH-1.186 | 0.63 | [7] |
| Athens, Greece | Csa | ASV=0.034T_a_+0.0001G-0.086v-0.001RH-0.412 | 0.27 | [8] |
| Thessaloniki, Greece | Cfa | ASV=0.036T_a_+0.0013G-0.038v+0.011RH-2.197 | 0.51 |  |
| Milan, Italy | Cfb | ASV=0.049T_a_-0.0002G+0.006v+0.002RH-0.92 | 0.44 |  |
| Fribourg, Germany | Cfb | ASV=0.068T_a_+0.0006G-0.107v-0.002RH-0.69 | 0.68 |  |
| Kassel, Germany | Cfb | ASV=0.043T_a_+0.0005G-0.077v+0.001RH-0.876 | 0.48 |  |
| Cambridge, UK | Cfb | ASV=0.113T_a_+0.0001G-0.05v-0.003RH-1.74 | 0.57 |  |
| Sheffield, UK | Cfb | ASV=0.07T_a_+0.0012G-0.05v-0.003RH-0.855 | 0.58 |  |

* Based on Köppen climate classification. The 1^st^ letter B signifies dry, while C indicates temperate. The 2^nd^ letter includes S for semi-arid or steppe, w for dry winter, f for no dry season, and s for dry summer. The 3^rd^ letter indicates h for hot, a for hot summer, k for cold, and b for warm summer.

** ASV: actual sensation vote, G: global solar radiation (W/m^2^), T_a_: air temperature (°C), RH: relative humidity (%), v and WS: wind speed (m/s), SR: solar radiation intensity (W/m^2^), HR: absolute humidity (g/Kg air), T_mrt_: mean radiant temperature (°C).

**Table S2.** Comparative Analysis of ENVI-met's Applications for Heat Mitigation, Thermal Comfort, and Air Quality

|  | **Heat mitigation** | **Thermal comfort** | **Air quality** |
| --- | --- | --- | --- |
| **Purpose of use** | To model urban microclimates and assess the impact of GI on reducing air and surface temperatures in urban areas. It simulates temperature variations in both vertical and horizontal domains considering shading, multiple reflections from GI, buildings, and surfaces as well as scatter and diffuse reflections. | To simulate the effects of GI and urban settings on static pedestrian thermal comfort by employing indices such as UTCI, PET, and other thermal comfort metrics. It also incorporates skin temperature changes, sweat rate, and other personal factors of a virtual pedestrian. | To model the dispersion and deposition of pollutants around GIs. It considers both particulate and gaseous components. It evaluates the effect of GI, urban settings, and pollution sources on concentrations of pollutants such as PM_2.5_ and NO_2_. |
| **Scale of application** | Based on literature, from street-scale to neighbourhood scale, with larger scales feasible but constrained by computational limits. | | |
| **Model input and output** | Input: land surface characteristics, building geometries, GI types, meteorological data, etc.  Output: Temperature distribution, including heat fluxes and temperature gradients. | Input: Urban geometrics, GI types, meteorological conditions, etc.  Output: Thermal comfort indices such as PET and UTCI. | Input: pollutant emission sources, GI characteristics, meteorological parameters, urban geometrics, etc.  Output: pollutant concentration maps |
| **Strengths and limitations** | It can model complex interactions between urban surfaces and microclimate, providing insights into heat mitigation. However, its high computational demand restricts large-scale applications. | It enables detailed assessments of microclimatic variations and their effects on human comfort. Additionally, it simulates dynamic thermal comfort, providing insights into changes in skin temperature, cold/warm sensations or sweat rate, etc. However, it does not account the individuals real thermal perception and behavioural responses. | It excels in simulating near-source pollutant dispersion and deposition, providing insights into the efficacy of GIs. However, it simplistic the complex atmospheric chemical and biological processes affecting pollutant removal and transformation. |
| **Outcomes and application** | Providing guidance for urban planners to effectively implement different GI types and supporting environmental health policies. | | |

**
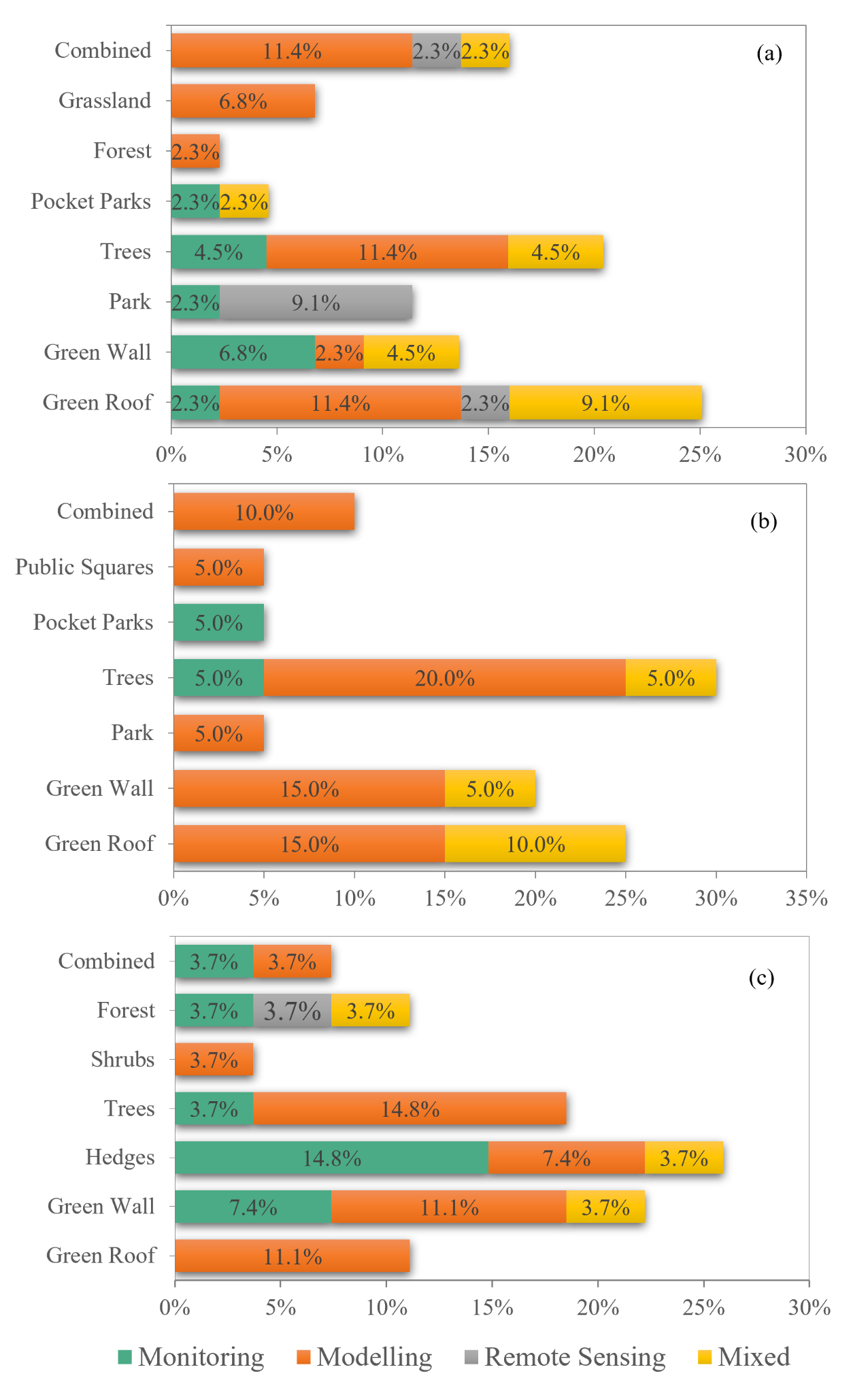
**

**Figure S1.** Distribution of (a) the cooling benefit, (b) thermal comfort, (c) air pollution studies on different GI type**s** using different evaluation methods (monitoring, modelling, remote sensing, and mixed approaches).

**S1 Heat mitigation benefit**

The potential of GI for mitigating the UHI effect has been estimated through the methodologies detailed in section 5.1. The reviewed studies evaluated the performance of one or more GI types in different regions. The growing number of papers in this field reveals the high importance of GIs as a practical method for reducing the harmful effects of UHI.

Quyang et al. (2021) tried to fill the gap of evaluating a single type of GI by investigating three GI typologies within one site, in addition to comparing their thermal irradiate performance for four typical summer days in Hong Kong [9]. Herath et al. (2018) ran a field assessment and simulation study in tropical Colombo, Sri Lanka to examine the cooling effects of tree curbsides, green roofs, green walls, and a combination of these GIs. The results revealed that the combination of all options is the best strategy for temperature reduction followed by trees in curbsides and green walls [10]. Jia and Wang (2021) evaluated different cooling strategies in a typical high-density urban area in Hong Kong highlighting the benefits of street trees in reducing both the air temperature and mean radiant temperature compared to urban cooling strategies which reduce the air temperature but resulting in increased mean radiant temperature [11]. The nature-based solution for urban heat mitigation in historical blocks of Beijing, China was investigated by simulating five different scenarios concluding that selecting proper GIs can reduce air temperature by 1.01 °C [12] .

Through assessing different GI scenarios for Guildford, UK, Tiwari et al. (2021) concluded that the trees are more effective compared with grasslands in reducing the UHI effects [13] . Ziaul and Pal (2020) examined the implication of GI on enhanced microclimatic conditions to evaluate the most suitable mitigation strategy for West Bengal, India. They evaluated the GI cooling effect in different urban areas, finding out that the effect of each GI type varies in different urban areas, and they concluded that the combination of all strategies is the most effective scenario for reducing UHI intensity [14].

Marando et al. (2019) carried out a study to find out the cooling capacity and ecosystem services of multiple types of GIs in Rome, Italy. The results clarified that the cooling capacity of GI depended on the GI element [15]. Through evaluating the effects of six different GI interventions on ambient temperatures across Portland, Oregon it was observed that results were inconsistent across landscapes revealing that different areas of the city require a site-specific application for mitigating UHI intensity [16].

Many studies have shown that re-integrating GIs into an urban landscape is an effective technique for urban climate mitigation and adaptation. However, the influence of GIs on temperature regulation varies greatly depending on GI type, species, and urban morphology [17-20]. Imran et al. (2019) evaluated the effectiveness of multiple urban vegetation patches in Melbourne during one of the most severe heatwave events concluding that the increased fraction of vegetated patches results in reducing UHI intensity during the night without a cooling effect for the near-surface temperature during the hottest time of the day [21]. Wang et al. (2021) found that increasing the green cover to 10% by adding street trees and grove reduces the UHI intensity in Guangzhou, whereas adding a green roof aggravated it [22]. The evaluation of GIs and urban settings as complex dynamic systems in Sydney, Australia confirms the effect of GI on LST reduction in both summer and winter while the results showed an increase of nocturnal LST throughout the year [23].

According to the findings of a study conducted in Wuhan, China, some parks had no cooling effect, whereas others had a cooling effect of up to 10°C LST reduction [17]. Therefore, although some studies recommend the most effective types of GIs in different climates, the selection of appropriate GIs remains dependent on local climatic conditions, soil features, and water availability in addition to some social behaviour in terms of community norms and cultural values [24-26]. As a result, the lack of a scientific basis for GI selection can lead to dysfunctional GIs in terms of urban heat mitigation so further studies need to consider the effects of different species and urban morphology on GIs’ effectiveness.

Implementing urban parks and some other types of GIs may not be practical in highly urbanised areas. Although urban ecosystems cannot be restored to their natural condition, green infrastructure such as pocket parks, green roofs, and green walls provide a realistic solution to heat flow regulation by absorbing incoming solar radiation. Green roofs and walls are frequently touted as a low-cost method of decreasing urban heat while also cooling buildings by insulating them and limiting energy transmission. Wang et al. (2022) evaluated the effectiveness of green roofs on UHI mitigation during a heatwave in Berlin confirming that green roofs affect the duration of thermal stress [27]. While some studies restricted the effectiveness of green roofs in the neighbourhoods, contributing to local temperature reduction [28] . Tan et al. (2014) quantified the effects of installing vertical greenery on the thermal quality of outdoor spaces in Singapore by monitoring the mean radiant temperature and air temperature at different distances of green walls. Although the air temperature did not fluctuate greatly at different intervals, the green wall had an effective impact on mean radiant temperature during the day and at night[29]. A previous study tried to present the concept of Green Structure (GS); its cooling efficiency is based on the synergy of shade and evapotranspiration for highly urbanised areas. A simple dynamic simulation of the GS performance clarified that the GS in a courtyard can result in an average potential reduction of between 17℃ and 29℃ in mean radiant temperature [30].

A recent study showed a slightly higher amount of air temperature inside Hong Kong’s pocket parks rather than outside the parks highlighting the effects of wind conditions and building geometry on outdoor air temperature. This study also suggests that the building shade may have a higher impact than greenery in the unique urban context of Hong Kong [18]. Park et al. (2021) compared the performance of building shade and greenspace in mitigating UHI intensity by monitoring six sunny days in six different blocks in Seoul, Korea clarifying that the cooling effect of greenspaces surpassed the building shade by up to 0.87℃ [31]. In order to increase the cooling effect of GIs, greenery should be combined with active strategies such as breeze enhancement and water spraying [32].

GIs can help to recover the previous character of the cities, each tree in the city mitigates additional heat exposure of one heat-sensitive person [33]. The effectiveness of GIs in cooling capacity is related to the original condition of the urban surfaces and the type of the injected GI as well as the intensity of it [34]. The benefits of using GIs vary across climate types with the greatest performance in hot and dry climates, up to around 12℃ [32]. To ensure healthy urban living conditions for dwellers it is necessary to consider that in almost 40% of the countries, a great portion of the residing population does not benefit from the microclimatic regulation provided by GI [35].

**S2 Thermal comfort**

The thermal comfort benefit offered by different GI types has been widely evaluated using the discussed methods. These studies delved into different aspects to measure the efficiency and identify influential factors across varying climate types to improve thermal comfort conditions in urban areas.

Balany et al. (2022) investigated several scenarios in the central business district of Melbourne, Australia. The results show that within the context of high-rise buildings, green roofs and green walls exhibit a modest decrease in the ambient temperature, up to approximately 0.47°C and 0.27°C, respectively, with no discernible enhancement in the perceived thermal comfort levels [36] . Chen and Ng (2013) analysed the thermal comfort conditions of two scenarios (50% of tree coverage and 30% of grass coverage) to assess pedestrian thermal comfort in the subtropical climate of Hong Kong. Based on the generated thermal confront maps, both tree and grass scenarios can decrease the mean PET of the domain by 0.4 K [37]. Lobaccaro and Acero (2015) evaluated the thermal comfort conditions in three distinct urban street canyons distinguished by varying geometric dimensions and under five different urban greenery scenarios on a typical summer day. The findings revealed that combining trees and grass, results in a reduction of two thermophysiological categories during peak daytime temperatures [38].

Cui et al. (2022) evaluated the effects of different vertical greenery arrangements with a certain amount of greenery. The results showed that vertical greenery can improve PET thermal comfort index by 0.17 °C to 1.4 °C. They concluded that the main factor which determines the pedestrians' thermal comfort condition is the coverage rate of greening facade at the pedestrian level [39] . Zhao et al. (2018) simulated various tree layouts (clustered, dispersed, and equal interval) in a neighbourhood to compare the outdoor thermal comfort conditions. They found out that an equal spacing of two trees yielded the greatest advantages in terms of microclimate improvement and human thermal comfort within the neighbourhood followed by a configuration involving clustered trees without overlapping canopies [18]. Kong et al. (2017) examined the influence of different trees on urban microclimate and outdoor thermal comfort in open spaces and high-density settings. The study reveals that trees in high-density settings have a more significant impact on enhancing pedestrian thermal comfort than those in open spaces. These studies highlight the importance of strategic implementation of GIs to enhance thermal comfort as an essential component of urban developments [41].

Furthermore, several previous studies highlighted the effect of physiological factors on thermal comfort conditions. The results of a field survey conducted in University of Groningen, Netherlands revealed that the four significant parameters affecting thermal comfort conditions were exposure time in GI, previous thermal environment and activity, as well as their thermal history. This study suggested that non-physical and subjective factors are more important in perceiving thermal comfort than the real thermal conditions [42]. A wide range of studies aimed to find the neutral PET range in different regions. Andrade et al. (2011) estimated the thermal comfort range of PET as 21°C to 23°C in Lisbon, Portugal [43] while the neutral range for Belo Horizonte is a wider range from 16°C to 30°C [44]. Another study conducted in Glasgow, UK determined the neutral PET range at a much lower range from 9°C to 18°C [45] . These findings highlight the effect of peoples’ expectations based on the climate type and other physiological factors that need to be considered in assessing the thermal comfort conditions.

**S3 Air quality**

Many studies aimed to estimate the concentration reduction of pollutants provided by different GI types. Through a field measurement in an urban park, Cohen et al. (2014) estimated the pollution mitigation benefit provided by a park during extreme events as up to 324 ppb NO_x_ and 56 mG/m^3^ PM_10_ , while the O_3_ values increased by 10 ppb [46]. Yin et al. (2011) found out that the studied park can contribute to 9.1% of total suspended particles removal, 5.3% of SO_2_, and 2.6% of NO_2_. They also highlighted the crown volume coverage and the pollution diffusion distance are the key predictors affecting the removal rate of the pollutants [47]. Pugh et al. (2012) investigated the interplay between urban form and vegetation, showing that enhancing the presence of vegetation within urban street canyons has the potential to decrease street-level concentrations in these areas significantly, with reductions of up to 40% for NO_2_ and 60% for PM [48] . Jia et al. (2021) found out that the existence of GI had a notable impact on particle concentrations over bike lanes and sidewalks. When compared to scenarios without GI, a reduction of up to 20% for BC and 28% for PM was observed on the paths after implementing GI [49]. Muresan et al. (2022) identified an area of approximately 121 hectares within the central urban zone of the Municipality of Ferrara, presenting a promising opportunity for afforestation which could lead to a remarkable enhancement in the removal of PM_10_ and O_3_ pollutants by approximately 49% and 18%, respectively [50].

Abhijith and Kumar (2019) evaluated the changes in pollutants concentrations behind and in front of GI during morning and evening traffic peaks. The results showed that the ‘hedges only’ scenario showed higher reductions among the two others ‘trees only’ and ‘a mix of trees and hedges/shrubs’ scenarios with up to 63% and 14% concentration reduction for Black Carbon and PM_2.5_ [51]. Kumar et al. (2022) evaluated the vertical and horizontal distributions of pollutants in a street canyon in West London. The results showed graded filtering of pollutants by GI and the distribution patterns around an evergreen roadside hedge depending on the wind direction [52]. Motie et al. (2023) aimed to find the optimal patterns of GI in canyons to reduce the air pollution. The findings reveal that coniferous trees on sidewalks have the least impact, while deciduous trees in the median strip can significantly enhance air quality. Shifting deciduous trees from sidewalks to the median strip reduced summer air pollution by 67% and winter pollution by 54% at pedestrian level [53] .

Kandelan et al. (2022) tried to find the optimised scenario within different patterns of GI on the sidewalks. Simulation results indicate a strong correlation between AQI rates, GI, and wind direction. In the optimised scenario, PM_2.5_ ambient concentrations can be reduced by up to 33% at the 1.75 m level [54]. Xu et al. (2023) explored the effects of GI on air quality from a three-dimensional perspective using street view imagery in Wuhan. They found out that street scale GIs greatly affect the air quality within 300m of the surrounding area. They indicated that high pollution levels, coupled with canyon effects, may intensify the adverse impact of street-scale GI on the dispersal of pollutants [55]. Another study within urban parks in Hong Kong investigated the impact of tree planting configurations on the diffusion of pollutants by examining the effect of tree morphology and landscape. The results showed that dense trees with a low crown base proved effective in enhancing air quality within parks when used as barriers, especially when planted at borders with a width of approximately 15 metres. However, excessive planting resulted in reduced wind speed and higher pollutant levels, highlighting the need to avoid overly dense arrangements and consider the tree distribution effect of airflow and pollutant dispersion [56].

GI can have both positive and negative effects on air pollution, highlighting the importance of selecting the most suitable assessment methods that considers all influential factors. On the positive side, well-designed GI can act as natural air filters, reducing pollutant concentrations and enhancing air quality. However, the impact of GI on air pollution can be negative if green elements are excessively planted or poorly positioned, hindering wind flow and trapping pollutants. Therefore, strategic consideration of influential factors is crucial to benefit from GI while minimising potential drawbacks, creating healthier and more sustainable urban environments.

**References**

[1] Aljawabra, F., & Nikolopoulou, M. (2018). Thermal comfort in urban spaces: a cross-cultural study in the hot arid climate. International journal of biometeorology, 62(10), 1901-1909.

[2] Yao, J., Yang, F., Zhuang, Z., Shao, Y., & Yuan, P. F., 2018. The effect of personal and microclimatic variables on outdoor thermal comfort: A field study in a cold season in Lujiazui CBD, Shanghai. Sustainable Cities and Society, 39, 181-188.

[3] Zhao, L., Zhou, X., Li, L., He, S., & Chen, R., 2016. Study on outdoor thermal comfort on a campus in a subtropical urban area in summer. Sustainable cities and society, 22, 164-170.

[4] Ruiz, M. A., & Correa, E. N., 2015. Adaptive model for outdoor thermal comfort assessment in an Oasis city of arid climate. Building and Environment, 85, 40-51.

[5] Lai, D., Zhou, C., Huang, J., Jiang, Y., Long, Z., & Chen, Q., 2014. Outdoor space quality: A field study in an urban residential community in central China. Energy and Buildings, 68, 713-720.

[6] Cheng, V., Ng, E., Chan, C., & Givoni, B., 2012. Outdoor thermal comfort study in a sub-tropical climate: a longitudinal study based in Hong Kong. International journal of biometeorology, 56, 43-56.

[7] Lai, D., & Chen, Q., 2012. Outdoor thermal comfort in northern China. Tianjin University.

[8] Nikolopoulou. M., 2004. Designing open spaces in the urban environment: a bioclimatic approach, Centre for Renewable Energy Sources, EESD, FP5.

[9] Li, Y., Ouyang, W., Yin, S., Tan, Z., & Ren, C. (2023). Microclimate and its influencing factors in residential public spaces during heat waves: An empirical study in Hong Kong. Building and Environment, 236, 110225.

[10] Herath, H. M. P. I. K., Halwatura, R. U., & Jayasinghe, G. Y., 2018. Evaluation of green infrastructure effects on tropical Sri Lankan urban context as an urban heat island adaptation strategy. Urban Forestry and Urban Greening 29, 212–222.

[11] Jia, S., & Wang, Y., 2021. Effect of heat mitigation strategies on thermal environment, thermal comfort, and walkability: A case study in Hong Kong. Building and Environment 201, 107988.

[12] Su, W., Zhang, L., & Chang, Q., 2022. Nature-based solutions for urban heat mitigation in historical and cultural block: The case of Beijing Old City. Building and Environment, 225, 109600.

[13] Tiwari, A., Kumar, P., Kalaiarasan, G., & Ottosen, T. B., 2021. The impacts of existing and hypothetical green infrastructure scenarios on urban heat island formation. Environmental Pollution 274, 115898.

[14] Ziaul, S., & Pal, S., 2020. Modeling the effects of green alternative on heat island mitigation of a meso level town, West Bengal, India. Advances in Space Research 65(7), 1789–1802.

[15] Marando, F., Salvatori, E., Sebastiani, A., Fusaro, L., & Manes, F., 2019. Regulating Ecosystem Services and Green Infrastructure: assessment of Urban Heat Island effect mitigation in the municipality of Rome, Italy. Ecological Modelling 392, 92–102.

[16] Makido, Y., Hellman, D., & Shandas, V., 2019. Nature-based designs to mitigate urban heat: The efficacy of green infrastructure treatments in Portland, Oregon. Atmosphere 10(5), 282.

[17] Chen, M., Jia, W., Yan, L., Du, C., & Wang, K., 2022. Quantification and mapping cooling effect and its accessibility of urban parks in an extreme heat event in a megacity. Journal of Cleaner Production 334, 130252.

[18] Lau, S. S., Lin, P., & Qin, H., 2012. A preliminary study on environmental performances of pocket parks in high-rise and high-density urban context in Hong Kong. International Journal of Low-Carbon Technologies 7(3), 215–225.

[19] Morakinyo, T. E., Ouyang, W., Lau, K. K. L., Ren, C., & Ng, E., 2020. Right tree, right place (urban canyon): Tree species selection approach for optimum urban heat mitigation - development and evaluation. Science of the Total Environment 719, 137461.

[20] Qiu, K. B., Jia, B. Q., & Cheng, J. F., 2017. Cool island effect of urban parks and its influencing factors within the Fifth Ring in Beijing. Chinese Journal of Ecology 36, 1984–1992.

[21] Imran, H. M., Kala, J., Ng, A. W. M., & Muthukumaran, S., 2019. Effectiveness of vegetated patches as Green Infrastructure in mitigating Urban Heat Island effects during a heatwave event in the city of Melbourne. Weather and Climate Extremes 25, 100217.

[22] Wang, Y., Ni, Z., Hu, M., Chen, S., & Xia, B., 2021. A practical approach of urban green infrastructure planning to mitigate urban overheating: A case study of Guangzhou. Journal of Cleaner Production 287, 124995.

[23] Bartesaghi Koc, C., Osmond, P., & Peters, A., 2018. Evaluating the cooling effects of green infrastructure: A systematic review of methods, indicators and data sources. Solar Energy 166, 486–508.

[24] Bowler, D. E., Buyung-Ali, L., Knight, T. M., & Pullin, A. S., 2010. Urban greening to cool towns and cities: A systematic review of the empirical evidence. Landscape and Urban Planning 97, 147–155.

[25] Norton, B. A., Coutts, A. M., Livesley, S. J., Harris, R. J., Hunter, A. M., & Williams, N. S. G., 2015. Planning for cooler cities: A framework to prioritise green infrastructure to mitigate high temperatures in urban landscapes. Landscape and Urban Planning 134, 127–138.

[26] Pataki, D. E., Carreiro, M. M., Cherrier, J., Grulke, N. E., Jennings, V., Pincetl, S., Pouyat, R. v., Whitlow, T. H., & Zipperer, W. C., 2011. Coupling biogeochemical cycles in urban environments: Ecosystem services, green solutions, and misconceptions. Frontiers in Ecology and the Environment 9(1), 27–36.

[27] Wang, X., Li, H., & Sodoudi, S., 2022. The effectiveness of cool and green roofs in mitigating urban heat island and improving human thermal comfort. Building and Environment, 217, 109082.

[28] Tiwari, A., Kumar, P., Kalaiarasan, G., & Ottosen, T. B., 2021. The impacts of existing and hypothetical green infrastructure scenarios on urban heat island formation. Environmental Pollution 274, 115898.

[29] Tan, C. L., Wong, N. H., & Jusuf, S. K., 2014. Effects of vertical greenery on mean radiant temperature in the tropical urban environment. Landscape and Urban Planning 127, 52–64.

[30] Bandurski, K., Bandurska, H., Kazimierczak-Grygiel, E., & Koczyk, H., 2020. The green structure for outdoor places in dry, hot regions and seasons-providing human thermal comfort in sustainable cities. Energies 13(11), 2755.

[31] Park, J., Kim, J. H., Sohn, W., & Lee, D. K., 2021. Urban cooling factors: Do small greenspaces outperform building shade in mitigating urban heat island intensity? Urban Forestry and Urban Greening 64, 127256.

[32] Huang, J., Hao, T., Wang, Y., & Jones, P., 2022. A street-scale simulation model for the cooling performance of urban greenery: Evidence from a high-density city. Sustainable Cities and Society 82, 103908.

[33] Venter, Z. S., Krog, N. H., & Barton, D. N., 2020. Linking green infrastructure to urban heat and human health risk mitigation in Oslo, Norway. Science of the Total Environment, 709, 136193.

[34] Abdulateef, M. F., & A. S. Al-Alwan, H., 2022. The effectiveness of urban green infrastructure in reducing surface urban heat island: Baghdad city as a case study. Ain Shams Engineering Journal 13, 101526.

[35] Marando, F., Heris, M. P., Zulian, G., Udías, A., Mentaschi, L., Chrysoulakis, N., Parastatidis, D., & Maes, J., 2022. Urban heat island mitigation by green infrastructure in European Functional Urban Areas. Sustainable Cities and Society 77, 103564.

[36] Balany, F., Muttil, N., Muthukumaran, S., Wong, M.S., & Ng, A.W.M., 2022. Studying the Effect of Blue-Green Infrastructure on Microclimate and Human Thermal Comfort in Melbourne’s Central Business District. Sustainability 14, 9057.

[37] Chen, L., & Ng, E., 2012. Simulation of the effect of downtown greenery on thermal comfort in subtropical climate using PET index: a case study in Hong Kong. Architectural Science Review 56, 297-305. [38] Lobaccaro, G., & Acero, J.A., 2015. Comparative analysis of green actions to improve outdoor thermal comfort inside typical urban street canyons. Urban Climate 14, 251-267.

[39] Cui, D., Zhang, Y., Li, X., Yuan, L., Mak, C.M., & Kwok, K., 2022. Effects of different vertical facade greenery systems on pedestrian thermal comfort in deep street canyons. Urban Forestry & Urban Greening 72, 127582.

[40] Zhao, Q., Sailor, D.J., & Wentz, E.A., 2018. Impact of tree locations and arrangements on outdoor microclimates and human thermal comfort in an urban residential environment. Urban Forestry & Urban Greening 32, 81-91.

[41] Kong, L., Lau, K.K-L., Yuan, C., Chen, Y., Xu, Y., Ren, C., & Ng, E., 2017. Regulation of outdoor thermal comfort by trees in Hong Kong. Sustainable Cities and Society 31, 12-25.

[42] Wang, Y., Groot, de Groot, R., Bakkwe, F., Wörtche, H., & Leenabs, R., 2017. Thermal comfort in urban green spaces: a survey on a Dutch university campus. International Journal of Biometeorology 61, 87-101.

[43] Andrade, H., Alcoforado, M-J., & Oliveira, S., 2011. Perception of temperature and wind by users of public outdoor spaces: relationship with weather parameters and personal characteristics. International Journal of Biometeorology 55, 665-680.

[44] Hirashima, S.Q., Da, S., Katzschner, A., Ferreira, D.G., de Assis, E.S., & Katzschner, L., 2018. Thermal comfort comparison and evaluation in different climates 23, 219-230.

[45] Kruger, E., Drach, P., Emmanuel, R., Corbella, O., 2014. Urban heat island ans differences in outdoor comfort levels in Glasgow, UK. Theoretical and Applied Climatology 112, 127-141.

[46] Cohen, P., Potchter, O., & Schnell, I., 2014. The impact of an urban park on air pollution and noise levels in the Mediterranean city of Tel-Aviv, Israel. Environmental Pollution 195, 73-83.

[47] Yin, S., Shen, Z., Zhou, P., Zou, X., Che, S., & Wang, W., 2011. Quantifying air pollution attenuation within urban parks: An experimental approach in Shanghai, China. Environmental Pollution 159, 2155-2163.

[48] Pugh, T., Mackenzie, A., Whyatt, J.D., & Hewitt, N., 2012. Effectiveness of Green Infrastructure for Improvement of Air Quality in Urban Street Canyons. Environmental Science and Technology 46, 7692-7699.

[49] Jia, Y-P., Lu, K-F., Zheng, T., Li, X-B., Liu, X., Peng, Z-R., & He, H-D., 2021. Effects of roadside green infrastructure on particle exposure: A focus on cyclists and pedestrians on pathways between urban roads and vegetative barriers. Atmospheric Pollution Research 12, 1-12.

[50] Muresan, A. N., Sebastiani, A., Gaglio, M., Fano, E. A., & Manes, F., 2022. Assessment of air pollutants removal by green infrastructure and urban and peri-urban forests management for a greening plan in the Municipality of Ferrara (Po river plain, Italy). Ecological Indicators 135, 108554.

[51] Abhijith, K. V, & Kumar, P., 2019. Field investigations for evaluating green infrastructure effects on air quality in open-road conditions. Atmospheric Environment 201, 132–147.

[52] Kumar, P., & Sharma, A., 2022. Assessing the outdoor thermal comfort conditions of exercising people in the semi-arid region of India. Sustainable Cities and Society, 76, 103366.[53] Motie, M.B., Yeganeh, M., & Bemanian, M., 2023. Assessment of greenery in urban canyons to enhance thermal comfort & air quality in an integrated seasonal model. Applied Geography 151, 102861.

[54] Kandelan, S.N., Yeganeh, M., Peyman, S., Panchabikesan, K., Eicker, U., 2022. Environmental study on greenery planning scenarios to improve the air quality in urban canyons. Sustainable Cities and Society 83, 103993.

[55] Xu, J., Liu, M., Chen, H., & Luo, M., 2023. Spatially heterogeneous influence of street greenery on street-level PM2.5 pollution using mobile monitoring from a three-dimensional perspective. Urban Climate 48, 101414.

[56] Xing, Y., Brimblecombe, P., Wang, S., & Zhang, H., 2019. Tree distribution, morphology and modelled air pollution in urban parks of Hong Kong. Journal of Environmental Management 248, 109304.
